# From Laboratory Screening to Field Application: A Comprehensive Review of Biocontrol Strategies Against Aflatoxin Contamination

**DOI:** 10.3390/toxins18100437

**Published:** 2026-10-11

**Authors:** Christopher Hernandez, Vincent Merlhiot, Selma P. Snini, Florence Mathieu, Vanessa Durrieu, Jean-Denis Bailly

**Affiliations:** 1 Laboratoire de Chimie Agroindustrielle, Toulouse INP, INRAE, University of Toulouse, 31400 Toulouse, France; christopher.hernandez@ensiacet.fr (C.H.); vanessa.durrieu@ensiacet.fr (V.D.); 2 École Nationale Vétérinaire de Toulouse, University of Toulouse, Toulouse CEDEX 3, 31076 Toulouse, France; vincent.merlhiot_19@envt.fr; 3 Laboratoire de Génie Chimique, Toulouse INP, CNRS, University of Toulouse, 31400 Toulouse, France; selma.snini@toulouse-inp.fr (S.P.S.); florence.mathieu@toulouse-inp.fr (F.M.); 4 École Nationale Vétérinaire de Toulouse, Laboratoire de Chimie Agroindustrielle, Toulouse INP, INRAE, University of Toulouse, 23 Chemin des Capelles, 31076 Toulouse, France

**Keywords:** aflatoxins, biocontrol, nonaflatoxigenic strains, microorganisms, essential oils, plant extracts

## Abstract

Aflatoxins are carcinogenic mycotoxins that can contaminate many crops and whose distribution is increasing due to climate change. Given the mutagenic and carcinogenic properties of aflatoxins, protecting public health requires minimizing dietary exposure. In the European Union, maximum regulatory levels can be as low as 0.10 µg/kg for AFB1 in processed cereal-based foods and baby foods. Once produced, aflatoxins are stable, so the best way to protect consumers is to minimize aflatoxin production. Numerous studies have investigated sustainable and eco-friendly strategies to limit aflatoxin contamination in food and feed. These strategies rely on microorganisms or natural, plant-derived compounds that hinder the development of aflatoxigenic species and/or specifically inhibit toxin biosynthesis. However, a substantial translational gap remains between initial in vitro evidence of the effects on fungal growth and/or aflatoxin synthesis and the effective application of such biocontrol agents under field conditions. This review aims to clarify the current state of the art by presenting the diverse biocontrol strategies currently being investigated to combat aflatoxins. We discuss the advantages and disadvantages of such strategies and describe the level of technological readiness, which spans from in vitro tests to large-scale in-field applications.

## 1. Introduction

Aflatoxins are toxic secondary metabolites produced by several species of fungi, most of which belong to the *Aspergillus* genus [1,2]. Although *Aspergillus flavus* and *A. parasiticus* are the main aflatoxin-producing species, other *Aspergillus* species, including *A. nomius*, *A. pseudotamarii*, *A. ochraceoroseus*, and *A. rambellii*, also produce aflatoxins [2,3]. These different species synthesize four molecules named aflatoxin B1 (AFB1), aflatoxin B2 (AFB2), aflatoxin G1 (AFG1), and aflatoxin G2 (AFG2). The characteristics of the toxin produced depend on the species; *A. flavus* produces only B-type aflatoxins, whereas *A. parasiticus* simultaneously produces B- and G-type aflatoxins. Moreover, the ability to produce the toxin depends on the fungal strain. In *A. flavus* populations, toxigenic and atoxigenic isolates coexist, so the aflatoxin-producing capacity cannot be inferred from species identity alone. These molecules can be metabolized by animals after ingestion, leading to the formation of various derivatives. For example, aflatoxin M1 (AFM1) and aflatoxin M2 (AFM2) are hydroxylated metabolites produced in the liver following the ingestion of AFB1 and AFB2, respectively. These compounds can subsequently be excreted in milk [4].

The consequences of aflatoxin exposure depend on the type, duration, and dose of the exposure [5]. AFB1 is a known hepatotoxic compound [6,7,8]. Although liver damage is predominant upon exposure, other deleterious effects have been observed in both animals and humans, such as immunosuppression and teratogenic effects, as well as a decrease in feed intake, average daily gain, and reproductive success [5,6,9,10,11,12].

AFB1 is a major global public-health concern because of its well-established carcinogenicity in humans and its frequent occurrence across food commodities [13]. It is considered one of the most potent naturally occurring carcinogens and one of the natural foodborne contaminants that contribute most to disability-adjusted life years (DALYs) [6]. 

Aflatoxins are highly stable compounds and have low sensitivity to conventional thermal treatments such as sterilization, pasteurization, or freezing, or to dehydration or freezing processes (with the notable exception of roasting). Consequently, once contamination occurs, it is extremely difficult to detoxify food products by degrading the toxins in the food [6].

Prevention of toxin formation is therefore a central component of aflatoxin management. Preventive interventions can limit fungal establishment (e.g., by suppressing the biosynthesis of aflatoxin) or limit growth, or both [14,15]. The relationship between fungal contamination and aflatoxin production is complex because aflatoxins are secondary metabolites whose biosynthesis depends on numerous factors, including cell signaling, growth conditions, reproductive processes, and oxidative stress [1]. Therefore, understanding the life cycle of these fungi is essential for controlling their development and minimizing mycotoxin contamination in agricultural products, allowing for more precisely targeted preventive actions [15]. Within the genus *Aspergillus*, aflatoxigenic species are predominantly saprotrophic but can also behave as opportunistic plant pathogens [15,16,17]. Indeed, plant contamination often requires a decline in plant vigor, which may occur under conditions such as heat or drought that cause abiotic stress. Aflatoxigenic species are thermophilic and relatively xerophilic [15]. Tropical and subtropical regions, particularly between 26° and 35° latitude north and south, often provide favorable conditions for fungal growth and aflatoxin production [18]. However, climate change is expanding this favorable zone into regions previously considered “aflatoxin-free,” notably into southern Europe, including France [1,15,19,20,21].

Aflatoxins are capable of contaminating a wide variety of plant-based raw materials. They have been detected in cereals (e.g., maize, sorghum, wheat), oilseeds (e.g., almonds, hazelnuts), figs, tobacco, and many other commodities [2,4,22]. Milk can also be contaminated following the ingestion by dairy animals of aflatoxin-contaminated feed and the subsequent milk excretion of the hydroxylated derivatives (e.g., AFM1) [23]. Due to the high consumption levels of cereals and milk, these are the primary contributors to human exposure to these mycotoxins. In particular, maize and its derived products contribute strongly to AFB1 exposure, and milk and milk powder contribute strongly to AFM1 exposure [6]. 

Aflatoxin contamination occurs throughout the entire production cycle of plant-based raw materials: in the field, at harvest time, and during storage—particularly given insufficient drying or if re-moistening occurs [6]. In the field, contamination may be facilitated by infestations of boring insects, which provide easier access to the plant’s internal nutrient-rich structures for mold colonization [6]. Periods of water stress also promote contamination [4,24,25]. *Aspergillus* fungi can survive on plant debris as sclerotia (overwintering survival structures composed of compacted mycelium), conidia, or hyphae [15,26], with soil being the primary inoculum. Conidia already present in the soil or on crop residues, as well as conidia produced following sclerotia germination, can be dispersed directly by wind and insects [15].

Contamination pathways depend on the type of crop. For example, the harvested parts of peanuts are in direct contact with the soil, so contamination occurs primarily at ground level. Entry points for fungal invasion may be created by insect damage or climatic conditions—for example, drought can lead to cracks in peanut pods, facilitating colonization by fungi [15,27]. In contrast, the harvested part of maize is aerial, so contamination typically occurs during anthesis. The silks serve as an entry route through which fungi reach the developing ear. Additionally, injuries caused by insect activity facilitate contamination [26,27]. Insects may deposit inoculum directly if they are carrying spores, or they may create lesions in the kernels that allow the penetration of windborne spores [15,28]. At harvest, dust, soil, and plant debris from previous crops can also contribute to cereal contamination [6].

Due to the adverse health effects of aflatoxins, dietary exposure should be minimized. Regulatory authorities have therefore established maximum or action levels for AFB1 and/or total aflatoxins in food. However, these limits are not globally harmonized but vary according to the country, the aflatoxin considered, the type of food commodity, the intended use or processing stage of foodstuffs, and the target consumer population. As summarized in Table 1, regulatory approaches also differ: some regulations establish separate limits for AFB1 and total aflatoxins, whereas others regulate only one of these parameters. Consequently, a single threshold cannot accurately represent aflatoxin regulations worldwide.

To satisfy these regulatory requirements and minimize consumer exposure, multiple strategies have been developed to prevent or reduce aflatoxin contamination throughout the food chain. These strategies include biological, chemical, and physical control methods; however, all approaches begin with and rely on the implementation of good agricultural practices (GAPs). The implementation of GAPs is essential and constitutes the first line of defense against fungal contamination. In addition to improving food safety and quality, GAPs also enhance crop yield and reduce postharvest losses, making them doubly advantageous. These practices can be applied at all stages of crop production—preharvest, harvest, and postharvest—and several studies have summarized the strategies applicable at each stage [34]. In the field, GAPs pursue four main objectives: (i) limit the establishment and persistence of fungi; (ii) restrict the dissemination of fungi; (iii) reduce the development of fungi and plant contamination by fungi; and (iv) enhance plant resistance [15,35,36,37,38].

Although these practices are necessary, they do not always suffice, and complementary sustainable and environmentally compatible control methods remain necessary. Because the term “biocontrol” is used inconsistently across the aflatoxin literature, we adopt herein a broad operational definition that encompasses live antagonists, nonviable microbial products, and plant-derived substances used to prevent fungal development and/or aflatoxin formation. Studies limited to adsorption, binding, or transformation of preformed aflatoxins are considered only when required to distinguish decontamination and detoxification from preventive control. Within this scope, the following three categories are examined:

(i) Atoxigenic strains of *A. flavus* that competitively exclude toxigenic counterparts;
(ii) Other microorganisms and their cell-free products or metabolites with antifungal or antiaflatoxigenic activity;
(iii) Plant-derived substances, including essential oils, extracts, and purified compounds.

Each of these methods targets different stages of the fungal life cycle or of toxin biosynthesis and offers specific advantages and disadvantages. The following sections provide a comprehensive overview of how these biocontrol agents have been developed, their mechanisms of action, and the possible current barriers to their implementation on a larger scale.

Several recent reviews have addressed broad aflatoxin detection and mitigation, global microbial research trends, actinobacterial biocontrol, selected biological strategies, native atoxigenic *Aspergillus*, nano-formulated essential oils, and microbial or enzymatic detoxification of preformed aflatoxins [39,40,41,42,43,44,45,46]. 

Taken together, these reviews reveal substantial advances within individual approaches, but no single review has critically integrated live microbial agents, cell-free microbial products, and plant-derived substances within a common development framework spanning laboratory screening, food matrices, greenhouse and field verification, formulation, stability, scale-up, safety, regulation, and commercial readiness. The present review addresses this gap by distinguishing (i) inhibition of fungal development, (ii) specific suppression of aflatoxin biosynthesis, and (iii) decontamination and detoxification of preformed aflatoxins, while focusing primarily on preventive interventions. Comparing evidence at progressively more realistic scales allows us to identify not only which candidates are active, but also why some approaches—most notably those involving native atoxigenic *A. flavus* strains—have progressed to the stage of registered products, whereas many bacterial, fungal, yeast, cell-free, and plant-derived candidates remain at the in vitro or proof-of-concept stage.

Accordingly, the comparative assessment applied throughout this review considers not only the magnitude of the reported antifungal or antiaflatoxigenic activity, but also the consistency and maturity of the evidence across target matrices, controlled plant experiments, field trials, formulation, safety evaluation, regulatory assessment, and commercial implementation.

**Literature Search and Selection Approach.** This comprehensive narrative review is based on a targeted and iterative literature search conducted using Scopus and Google Scholar. No lower publication-date limit was applied, and the search was updated through September 2026. Publications written primarily in English were considered. Representative search terms included “aflatoxin,” “aflatoxin B1,” “AFB1,” “aflatoxigenic,” “*Aspergillus flavus*,” “biocontrol,” “biological control,” “atoxigenic strain,” “nonaflatoxigenic strain,” “bacteria,” “yeast,” “filamentous fungi,” “cell-free supernatant,” “microbial metabolite,” “essential oil,” “plant extract,” “natural compound,” “antifungal,” and “antiaflatoxigenic.” These terms were combined, as appropriate, with terms related to the development and practical implementation of biocontrol strategies, including “food matrix,” “greenhouse,” “field trial,” “formulation,” “encapsulation,” “stability,” “scale-up,” “biosafety,” “regulation,” and “commercialization.” Additional relevant publications were identified from the reference lists of key original studies and recent reviews.

Original research articles were included when they evaluated the preventive effects of atoxigenic *A. flavus* strains, other microorganisms and their cell-free products or metabolites, or plant-derived substances against aflatoxigenic fungi or aflatoxin production. Relevant review articles and regulatory or technical documents were also considered when needed to address mechanisms of action, formulation, biosafety, field application, regulation, and commercialization. Studies dealing exclusively with the physical or chemical removal of preformed aflatoxins were outside the primary scope of the review and were not considered. Studies limited to the adsorption, binding, or transformation of preformed aflatoxins were considered only when required to distinguish detoxification from preventive biocontrol. Publications were selected according to their relevance and contribution to the objectives of this comprehensive narrative review rather than according to a formal systematic-review protocol.

## 2. Development of a Biocontrol Agent: From Laboratory to Field Applications

The development of a successful biocontrol strategy targeting aflatoxin contamination is a multistep process that typically progresses from basic laboratory assays to full-scale field verification. Regardless of their nature (whether microorganisms, atoxigenic fungal strains, or plant-derived substances), biocontrol agents share a common developmental pathway characterized by five key stages: initial screening, target-matrix verification, formulation development, efficacy testing under controlled and real conditions, and application optimization. Each step presents specific scientific and practical challenges that must be addressed to ensure the safety, efficacy, and scalability of these interventions.

**Figure 1:**
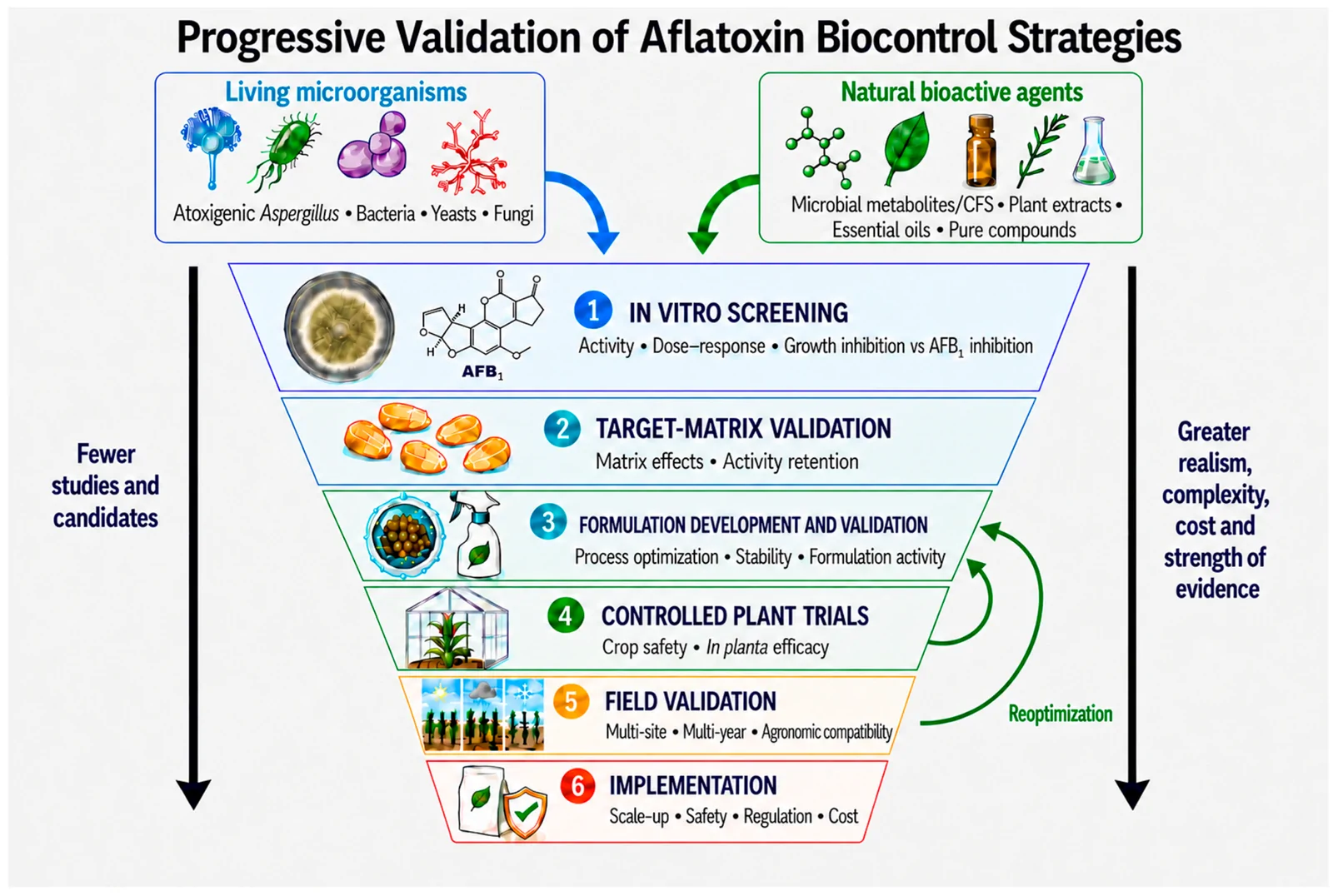
Conceptual framework for the progressive verification of aflatoxin biocontrol strategies from laboratory screening to implementation. This original figure was created by the authors and was not adapted from previously published material.

Figure 1 provides a conceptual overview of this progressive and iterative development pathway, and the following subsections discuss the principal scientific and technical considerations involved across the various stages of development.

### 2.1. Initial Screening and Selection

The first step involves the identification and selection of potential biocontrol candidates from the natural environment. For microorganisms, this means isolating strains from soil, rhizospheres, or plant surfaces, followed by morphological, biochemical, and molecular characterization. In this step, we should consider the origin and nature of the potential biocontrol agent, its abundance in its natural habitat, its ability to grow or act in different environmental conditions, and the mechanisms underlying its antifungal or antimycotoxin activity [47].

In the case of atoxigenic strains, the first step involves identifying local atoxigenic strains. The use of local strains appears to be essential because they are better adapted to the environment and more competitive against local aflatoxin-producing strains. Furthermore, the presence of atoxigenic strains in the environment is not uncommon, which provides an additional argument against the introduction of “foreign” strains into cropping systems [48]. A set of strains isolated from the environment is morphologically characterized, and its ability to produce mycotoxins as well as its membership in a vegetative compatibility group (VCG) is determined using microbiological, chemical, and molecular tools. Within a VCG, the ability of strains to produce aflatoxins is generally homogeneous [48,49,50,51,52]. Some VCGs are composed exclusively of atoxigenic isolates, making them particularly suitable candidates as biocontrol products [48,53,54].

The natural substances derived from plants, natural molecules, and extracts with fungicidal or antimycotoxigenic effects are generally divided into three categories: essential oils, crude extracts, or purified compounds [3]. Since the chemical nature of plant-derived molecules may vary greatly, the various extraction conditions are commonly screened, followed by chemical characterization to identify compounds with desirable properties.

Numerous candidates are eliminated at this stage. For example, in a screening of over 11,000 bacterial isolates, only three showed sufficient antifungal and antimycotoxigenic potential in in vitro and grain assays [47]. 

### 2.2. Characterization of the Mechanism of Action

Understanding how biocontrol agents inhibit fungal development and/or suppress aflatoxin biosynthesis is crucial for optimizing their performance and ensuring public-health safety. Gaining this knowledge may involve various strategies, which are now discussed.

A primary strategy is to reduce the yield of aflatoxin biosynthesis, which is often linked to interference in gene expression or metabolic pathways. In *A. flavus*, the genes directly involved in the successive enzymatic steps of aflatoxin biosynthesis are mainly grouped within a cluster of approximately 30 genes spanning about 70–75 kb on chromosome III [1,55]. These genes encode various enzymes that catalyze the conversion of precursor molecules (acetyl-CoA and malonyl-CoA) into aflatoxins [55]. This biosynthetic process involves more than 20 successive enzymatic reactions [48,56,57]. The genes within this cluster can be affected by various types of mutations, including substitutions, insertions, deletions, and frameshift mutations, all of which may hinder the production of aflatoxins [48,58,59]. They can also be affected by external factors such as the use of molecules with biological activities (e.g., antioxidant activity) [1,3].

Another strategy involves investigating the activity of volatile and nonvolatile compounds, enzymes, or signaling molecules. Volatile organic compounds (VOCs), such as alcohols, ketones, or terpenes, can inhibit fungal growth remotely through atmospheric diffusion [60]. In contrast, nonvolatile metabolites including organic acids, lipopeptides, and phenolic compounds typically act through direct contact or membrane disruption [61]. Enzymes such as chitinases, glucanases, and proteases may degrade fungal cell walls or interfere with key physiological processes, whereas microbial or plant-derived signaling molecules modulate fungal gene expression, including the downregulation of aflatoxin biosynthetic pathways [47]. Understanding these mechanisms not only enhances the rational selection of potent biocontrol agents but also informs strategies to improve their stability, delivery, and synergistic use.

Finally, assessing the potential for synergistic effects when combining agents (e.g., microbial consortia or multicompound plant extracts) is a critical step in biocontrol development because interactions between various biological agents can significantly enhance efficacy beyond that obtained from individual treatments. In microbial consortia, different strains may complement each other by targeting distinct steps in the pathogen’s life cycle, producing diverse antifungal compounds, or improving colonization of the plant or substrate. For instance, combinations of *Pseudomonas* and *Bacillus* strains have shown superior inhibition of *A. flavus* and aflatoxin biosynthesis compared with single strains, highlighting their synergistic potential [47]. Similarly, plant extracts composed of multiple bioactive molecules such as terpenes, phenolics, and aldehydes often demonstrate enhanced antifungal activity through additive or synergistic interactions acting on different cellular targets. Studies have shown that mixtures of essential oils or crude extracts from *Ocimum basilicum* and other species are more effective than isolated constituents [60,61]. 

Evaluating these combinations requires careful experimental design to distinguish between additive, synergistic, or antagonistic effects, and to ensure that the agents remain stable, nonphytotoxic, and compatible with agricultural practices.

### 2.3. Efficacy Testing in Controlled Conditions

Once promising biocontrol candidates are identified, their effectiveness must be evaluated under progressively more realistic conditions that mimic the natural environment and target crop systems. This staged approach includes in vitro assays, in situ tests on food matrices, and greenhouse or growth chamber experiments, each of which provides key insights into the performance and limitations of the agent prior to field deployment.

#### 2.3.1. In Vitro Assays

Initial assessments are often conducted on synthetic media (e.g., PDA, YES) using dual culture, agar diffusion, or microplate assays. These tests measure direct antifungal activity (e.g., inhibition zones, reduction in mycelial growth) or the ability to reduce aflatoxin production under controlled conditions [3,47,61,62].

#### 2.3.2. In Situ Assays on Food Matrices

Because synthetic media lack the complexity of real substrates, efficacy must also be tested on naturally susceptible commodities, such as maize kernels, peanuts, or dried fruit. These assays simulate contamination scenarios by inoculating the substrate with *Aspergillus spp*. and applying the biocontrol agent under controlled humidity and temperature. For example, García-Díaz et al. (2019) evaluated niosome-encapsulated *Satureja montana* and *Origanum virens* essential oils in artificially contaminated maize grain and reported reductions in *A. flavus* growth and aflatoxin accumulation during storage [63]. Such assays are crucial to assess the stability and adherence of the biocontrol agent and any possible interactions with food components that may inhibit or enhance its efficacy.

#### 2.3.3. Greenhouse and Growth Chamber Experiments

Greenhouse trials bridge the gap between lab and field by introducing live plants and more complex ecological interactions. Biocontrol agents are applied during key phenological stages (e.g., flowering, grain filling), and infection is simulated through artificial inoculation with toxigenic strains. Such setups allow researchers to assess not only antifungal efficacy, but also plant compatibility, potential phytotoxicity, and interaction with beneficial microbes. Examples include the greenhouse evaluation of the native nonaflatoxigenic *A. flavus* isolate CS1 in maize [64] and the assessment of a bioplastic-based seed coating containing *A. flavus* NRRL 30797 under greenhouse and field conditions [65].

### 2.4. Field Trials and Environmental Evaluation

Field trials are essential for verifying the efficacy and reliability of biocontrol agents under real agricultural conditions. Unlike laboratory or greenhouse studies, field experiments expose biocontrol agents to a wide range of environmental variables such as temperature fluctuations, UV, wind, rainfall, and competition with native microbiota [66,67,68]. These factors can influence fungal development, the establishment of biocontrol agents, and treatment efficacy. Moreover, the variation in environmental conditions between cropping seasons means that multiyear field trials are necessary to assess the reproducibility and robustness of biocontrol efficacy. Consequently, laboratory assays should be considered an initial screening criterion rather than sufficient evidence of field efficacy.

The Italian atoxigenic *A. flavus* strains provide a well-documented example of why field trials are necessary. In viable maize kernel assays, all 18 Italian atoxigenic isolates tested significantly reduced AFB1 contamination caused by the toxigenic strain AF13, with reductions ranging from 61% to 90%. Nine of the 18 isolates achieved reductions exceeding 80%, and five were identified as the most effective candidates. Among these candidates, A2085 and A2321 were further evaluated against six Italian aflatoxin-producing isolates. Both were effective in these laboratory assays, reducing AFB1 contamination by 60–78% and 61–83%, respectively [69].

Unfortunately, the two strains differed when evaluated on developing maize ears under field conditions. In pin-bar co-inoculation trials conducted in 2012 and 2013, A2321 did not significantly reduce AFB1 contamination relative to inoculation with the toxigenic strain A2092 alone. In contrast, A2085 reduced AFB1 concentrations by 93.2% and 98.3% in 2012 and 2013, respectively [70]. Thus, of the two laboratory candidates subjected to this direct field comparison, only A2085 retained significant efficacy.

A broader selection process was reported in Ghana. All 12 atoxigenic *A. flavus* isolates selected from 847 genotyped candidates strongly reduced AFB1 and AFB2 accumulation in laboratory assays. Although the three four-isolate formulations were also effective in maize and groundnut fields, individual recovery revealed marked differences in crop adaptation. Notably, GHM173-6, the most effective isolate in the laboratory, showed the poorest field recovery, whereas GHM511-3 combined strong laboratory inhibition with high recovery from both crops. Based on their field occurrence, competitive potential, and adaptation, eight candidates were ultimately selected for Aflasafe GH01 and GH02 [52]. Because the isolates were applied as mixtures, field efficacy was demonstrated at the formulation level, whereas individual selection relied primarily on their establishment and recovery.

A similar selection process occurred in Nigeria. A four-strain formulation reduced combined AFB1 and AFB2 contamination in maize by 57.2–99.2% across four agroecological zones over two years. However, La3303 was recovered much less frequently from treated grain (4.3–10.5%) than the other strains (16.7–32.1%). The authors therefore considered it an inefficient competitor and recommended its exclusion from subsequent formulations [71]. This result shows that field evaluation can identify unsuitable components even when the overall formulation is effective in laboratory assays.

Together, these studies illustrate complementary filters that arise in the development of biocontrol agents. The Italian experience [69,70] provides direct evidence that efficacy in laboratory kernel assays does not necessarily predict significant aflatoxin reduction in the field. The Ghanaian and Nigerian studies further show that, even when a formulation is effective, its constituent isolates may differ in establishment, crop adaptation, and competitive ability. Consequently, the selection of final candidates should consider both aflatoxin reduction and the ecological performance of each biocontrol agent in the target agroecosystem.

### 2.5. Formulation and Optimization of the Application Procedure 

The success of a biocontrol agent in the field depends not only on the agent itself but also on how it is formulated and applied. After field verification, a promising biocontrol agent must be converted into a product that retains its efficacy during formulation, storage, transport, and application. This scenario introduces additional selection criteria because the performance of the final product cannot be inferred solely from that of the unformulated active agent. Each carrier, combination, and application protocol must therefore be evaluated independently.

#### 2.5.1. Formulations Containing a Single Biocontrol Active Agent

Some formulations contain a single biocontrol active agent, such as a microbial strain, CFS, plant extract, essential oil, or purified compound, together with the carriers, protectants, or stabilizers required for its delivery. For live microorganisms, such formulations must preserve viability and enable the release of viable units (e.g., spores or cells) to allow their establishment in the target environment. For plant-derived substances, these formulations must limit the loss of activity caused by factors such as volatility or degradation.

For example, bioplastic granules provide a stable carrier for the atoxigenic *A. flavus* strain NRRL 30797 and support its establishment in soil [72]. For plant-derived active biocontrol agents, spray-drying an aqueous stem-bark extract of *Mimosa tenuiflora* with starch or maltodextrin preserves its dose-dependent inhibition of AFB1 production after one year of storage at room temperature. In contrast, the maximum AFB1 inhibition decreased from 65.7% to 43.8% when the unencapsulated extract was stored at −20 °C, representing a 33.3% decrease in its initial inhibitory activity. In contrast, soy-protein-based microparticles have a lower initial anti-AFB1 activity, demonstrating that the choice of wall material can directly affect the performance of formulations [73]. Similarly, encapsulation of the essential oils *Satureja montana* and *Origanum virens* in niosomes prolongs their activity against *A. flavus* in stored maize for up to 75 days [63]. These examples demonstrate that the formulation constitutes a new selection step: an effective biocontrol active agent can progress only if a suitable delivery system preserves its activity under the intended conditions of use.

#### 2.5.2. Formulations Containing Multiple Biocontrol Agents

Formulations may contain several microbial strains, different microbial species, multiple extracts or compounds, or combinations of microorganisms and plant-derived substances. However, the performance of such formulations cannot be predicted solely from the efficacy of their individual components.

Although the Ghanaian and Nigerian multistrain formulations discussed above effectively reduce aflatoxin contamination, their constituent isolates differ markedly in their establishment and recovery in the field, which influences the final inclusion or exclusion of individual strains [52,71]. Interactions between plant-derived substances can also depend on the exact composition of the mixture. Binary combinations of cinnamon, oregano, and lemongrass essential oils produce additive activity against *A. flavus*, whereas the three-oil mixture is synergistic only at a specific 1:5:48 ratio [74].

Thus, adding active agents introduces further variables, including component identity, proportion, contribution, and field persistence. As the number of components increases, so does the number of formulations requiring verification, and components that are effective individually may be excluded from the final optimized mixture.

### 2.6. Application Strategy and Commercial Feasibility

Once the active composition and carrier are selected, the application protocol creates an additional filter. Dose, timing, frequency, and application method determine whether the biocontrol agent reaches and remains in the target environment. In 14 maize field trials conducted over three years, early application of atoxigenic *A. flavus* products at the V5 growth stage generally proved the most effective strategy [75]. Similarly, although Aflasafe active ingredients persist after treatment, their frequency and efficacy decline when annual applications are discontinued, making application in each cropping season the most reliable recommendation under the conditions of evaluation [76].

Commercialization also requires reproducible production and adequate shelf life. When applied to approximately 19,000 ha of maize and groundnut, products manufactured using dried atoxigenic *A. flavus* spores were as effective as conventionally manufactured products [77]. More recently, atoxigenic-strain products retained sufficient sporulation capacity after four years of storage and after short-term exposure to 54 °C [78]. Although these results are product-specific, they demonstrate that manufacturing, storage, and transport conditions must be experimentally verified. Consequently, only formulation-application systems that remain effective and satisfy practical production and distribution requirements can progress to commercialization.

## 3. Use of Microorganisms as Biocontrol Agents

The use of microorganisms to control aflatoxin contamination has attracted increasing interest as a sustainable and eco-friendly alternative to chemical fungicides. This approach is based on harnessing the natural antagonistic capabilities of certain microorganisms such as bacteria, filamentous fungi, and yeasts to inhibit the growth of *A. flavus* or suppress aflatoxin biosynthesis. These microbial agents may act individually or in consortia, and their effectiveness depends on their adaptability to environmental conditions, colonization capacity, and specific mode of action [47].

### 3.1. Mechanisms of Action

The mechanisms of action of microorganisms can be divided into four main categories: competitive exclusion, antibiosis, mycoparasitism, and thigmoregulation and contact-dependent inhibition [79,80].

#### 3.1.1. Competitive Exclusion

This mechanism is the most well-documented for combating both general microbial antagonists and atoxigenic *A. flavus* strains. It involves biocontrol agents that colonize ecological niches more rapidly and efficiently than toxigenic strains, thereby limiting the latter’s access to space and nutrients [80,81]. In the case of atoxigenic strains, competitive displacement of toxigenic *A. flavus* has been repeatedly observed in field trials [82,83,84]. Hruska et al. (2014) showed that the reduction in aflatoxin contamination correlates positively with a decrease in the toxigenic fungal population and a simultaneous increase in the atoxigenic strain population [85].

However, the displacement mechanism alone does not fully explain all observed outcomes. For example, in poorly managed storage conditions, aflatoxin levels may still rise despite prior application of atoxigenic strains, suggesting the secondary growth of some toxigenic individuals or insufficient displacement [84,86]. Furthermore, in vitro studies show that aflatoxin suppression can exceed expected levels based on proportional strain replacement, suggesting more complex interactions [87,88].

#### 3.1.2. Antibiosis

Antibiosis refers to the production of bioactive compounds that directly inhibit fungal growth or aflatoxin biosynthesis, which includes the synthesis of lipopeptides, organic acids, VOCs, or other secondary metabolites. Table 2 summarizes some of the antifungal and anti-aflatoxigenic compounds identified from microbial sources.

In the context of atoxigenic strains, several studies suggest that they may produce VOCs or extracellular metabolites that inhibit aflatoxin biosynthesis in toxigenic strains. For instance, Moore, Lebar, and Carter-Wientjes (2022) [89] demonstrated that VOCs such as 2,3-dihydrofuran, 3-octanone, and decane produced by atoxigenic *A. flavus* strains reduce toxin production, most likely by inhibiting key biosynthetic enzymes or altering transcriptional regulation of the aflatoxin biosynthetic cluster. Similarly, cell-free filtrates from *A. flavus* atoxigenic or *A. oryzae* strains have been shown to suppress fungal growth, aflatoxin production, and gene expression in the aflatoxin biosynthesis cluster [90,91,92,93].

**Table 2:** Bacterial metabolites and enzymes associated with antifungal and/or antiaflatoxigenic activities.

Bacterial Producer (Strain)	Reported Metabolite(s), Enzyme(s), or Active Fraction	Reported Biological Role	Reference
*Achromobacter xylosoxidans* NFRI-A1	Cyclo(L-Leu-L-Pro)	Primarily antiaflatoxigenic; antifungal only at high concentration (>6 mg/mL)	[94]
*Bacillus amyloliquefaciens* A-1	Surfactin homologues ‡	Antifungal and antiaflatoxigenic	[95]
*Bacillus amyloliquefaciens* D-1	2,5-Dimethylpyrazine, phenethyl alcohol, 4-phenyl-2-butanone, 2,3,6-trimethylphenol, and 2,3,5-trimethylphenol †	Antifungal and antiaflatoxigenic	[96]
*Bacillus cereus* RQ13	2-Methylbutanoic acid, 9,12-octadecadienoic acid (Z,Z), cinnamic acid, and 3,4-dihydroxybenzoic acid, among others §	Antifungal and antiaflatoxigenic	[97]
*Bacillus licheniformis* BL350-2	3-Methyl-1-butanol §	Antifungal and antiaflatoxigenic	[98]
*Bacillus megaterium* BM344-1	Methyl hexadecanoate (methyl palmitate) and tetracosane §	Antifungal and antiaflatoxigenic	[99]
*Bacillus mojavensis* RC1A	Surfactin, iturin A, and fengycin ‡	Antifungal and antiaflatoxigenic	[100]
*Bacillus paramycoides* R2	Benzaldehyde and 3,7-dimethyl-1-octanol †	Antifungal and antiaflatoxigenic	[101]
*Bacillus safensis* TR-47	Dimethyl disulfide, 2-heptanone, 6-methyl-2-heptanone, and dimethyl trisulfide †	Antifungal and antiaflatoxigenic	[102]
*Bacillus siamensis* 3BS12-4	Protease, β-1,3-glucanase, and chitinase activities §	Antifungal	[103]
*Bacillus* spp. P1, P7, P11, and P45	Iturin A and surfactin homologues §	Antifungal	[104]
*Bacillus subtilis* BS-Z15	Mycosubtilin †	Antifungal and antiaflatoxigenic	[105]
*Bacillus subtilis* QST 713	Fourteen lipopeptides belonging to the iturin, fengycin, and surfactin families §	Antifungal and antiaflatoxigenic	[106]
*Bacillus velezensis* B2	Methylmalonic acid †	Antifungal and antiaflatoxigenic	[107]
*Bacillus velezensis* HC6	Iturin and fengycin †; surfactin §	Antifungal and antiaflatoxigenic	[108]
*Bacillus velezensis* ZH179; *Bacillus amyloliquefaciens* ZH409 and ZH99	Lipopeptide extracts containing surfactin, fengycin, iturin, and bacillomycin homologues ‡	Antifungal	[109]
*Flavobacterium johnsoniae* TR-18	Methyl thiolacetate, dimethyl disulfide, methyl isovalerate, 5-methyl-3-hexanone, and S-methyl 3-methylbutanethioate †	Antifungal and antiaflatoxigenic	[110]
*Klebsiella aerogenes* KTTM	Cyclo(L-Ala-Gly) †	Antiaflatoxigenic	[111]
*Lactiplantibacillus plantarum* ITEM 17215	Acetic acid, lactic acid, and 3-phenyllactic acid †; 1,2-dihydroxybenzene, benzoic acid, and p-hydroxyphenyllactic acid §	Antifungal	[112]
*Lactiplantibacillus plantarum* K35	2-Butyl-4-hexyloctahydro-1H-indene, oleic acid, palmitic acid, linoleic acid, and 2,4-di-tert-butylphenol, among others §	Antifungal and antiaflatoxigenic	[113]
*Lactiplantibacillus plantarum* UM55	Lactic, phenyllactic, hydroxyphenyllactic, and indole-lactic acids †	Antifungal and antiaflatoxigenic	[114]
*Lactiplantibacillus plantarum* WYH	Low-molecular-weight antifungal peptide fraction (AFPs-WYH) ‡	Antifungal and antiaflatoxigenic	[115]
*Pseudomonas fluorescens* PB27, *Bacillus cereus* B1, and *Bacillus thuringiensis* K1	Extracellular chitinolytic enzymes ‡	Antifungal	[116]
*Pseudomonas stutzeri* YM6	Dimethyl trisulfide †	Antifungal and antiaflatoxigenic	[117]
*Serratia marcescens* JPP1	Chitinase, including the characterized ChiBjp enzyme ‡	Antifungal and antiaflatoxigenic	[118,119]
*Stenotrophomonas* sp. strain no. 27 (closely related to *S. rhizophila*)	Cyclo(L-Ala-L-Pro) and cyclo(L-Val-L-Pro) †	Antiaflatoxigenic	[120]
*Streptomyces alboflavus* TD-1	Dimethyl trisulfide and benzenamine §	Antifungal and antiaflatoxigenic	[121]
*Vibrio gazogenes*	Prodigiosin †	Antiaflatoxigenic	[122]

† Individual compound or enzyme was purified, or an authentic or commercial standard was tested directly. ‡ Activity was demonstrated for an enriched or purified fraction, or the causal contribution of a metabolite class was supported by fractionation, biochemical, genetic, or metabolic evidence; however, all individual constituents were not tested separately. § Compounds or enzymes were detected in an active extract, supernatant, or volatile profile, but their individual contribution was not directly demonstrated. The inclusion of a bacterial strain or metabolite in this table does not imply regulatory authorization, demonstrated food or feed safety, or suitability for field application.

#### 3.1.3. Mycoparasitism

Mycoparasitism is the direct attack of one fungus by another. This interaction is mediated by genes involved in the synthesis of specific metabolites, primarily chitinases, glucanases, and proteases that enable the parasitic fungus to degrade and invade the host’s cells [80,123,124]. This mechanism of action is used by *Trichoderma harzianum*, which can parasitize toxigenic strains of *A. flavus* [125]. The biocontrol agent coils around the target hyphae of the host and eventually degrades them by entangling *A. flavus* hyphae with “infection pegs” [126]. Other fungi such as *Clonostachys rosea*, *Pythium oligandrum*, and *Sphaerodes mycoparasitica* also act through similar mechanisms [127,128,129,130].

#### 3.1.4. Thigmoregulation and Contact-Dependent Inhibition

Inhibition of aflatoxin production by atoxigenic strains may also involve thigmoregulation, which is a response to physical contact or mechanical stimulation. Huang et al. (2011), Raksha Rao, Vipin, and Venkateswaran (2020), and Sweany et al. (2022) all reported that the absence of direct contact between atoxigenic and toxigenic strains, when separated by a membrane, decreases aflatoxin production [131,132,133]. However, this effect depends on the aflatoxin strain, suggesting the involvement of additional or alternative mechanisms [133].

### 3.2. Application of Atoxigenic Strains

Atoxigenic strains are fungal strains that do not produce mycotoxins. These strains are used to compete against toxigenic strains in crops. The goal in the field is to limit the development of toxigenic strains or, at the very least, to reduce the production of the targeted mycotoxins by creating a form of “stress” that impacts secondary metabolism. After application, the atoxigenic biocontrol strains become predominant and subsequently limit the colonization of crops by toxigenic strains [2].

To control aflatoxins, the most effective and best-documented biocontrol strategy currently involves the use of nonaflatoxigenic strains. Research on their use began several decades ago with the pioneering work of Brown, Cotty, and Cleveland (1991) [134], who demonstrated the effectiveness of these strains in reducing cereal contamination. Today, numerous countries around the world are investigating this biocontrol approach (e.g., the United States, Nigeria, Kenya, Senegal, Ghana, Uganda, Gambia, Argentina, Mexico, Thailand, Malaysia, Italy, Serbia, Germany, Turkey). Studies assessing the effectiveness of nonaflatoxigenic strains are being conducted at various scales under controlled conditions (in vitro, on grains, or in greenhouses) and in field trials.

Generally, under controlled conditions, tests are carried out by co-inoculating nonaflatoxigenic with aflatoxin-producing isolates. Aflatoxin production is then quantified, and the reduction in contamination is calculated relative to controls where only aflatoxigenic isolates are introduced [48,69,135].

Field-scale verification is essential because the ability of fungi to compete with endemic strains depends on several factors, such as soil type and the availability of nitrogen, carbon, water, and minerals [136]. Plant defense responses against colonization by isolates—whether toxigenic or not may also play a role [137]. As a result, some isolates that were highly effective under controlled conditions later failed in open-field trials [52,70,71].

Trials conducted directly by farmers represent the final stage of testing [52,138,139,140]. In these cases, farmers apply the biocontrol strains using their habitual farming practices, maize varieties; and they do so on large plots of land. This stage is where the true effectiveness of the biocontrol agent is evaluated.

Table 3 presents the results reported in various studies conducted in different countries, under different conditions, and using different atoxigenic strains. Table 3 shows that the effectiveness of nonaflatoxigenic strains in reducing aflatoxin contamination varies widely (from 0% to 100%). This variability results from the wide diversity of strains tested (mainly *A. flavus* but also *A. parasiticus* and *A. tamarii*), the variety of experimental conditions, and the different types of studies conducted. However, in most cases, the use of atoxigenic strains significantly reduces the final aflatoxin contamination.

The heterogeneous results obtained in studies aiming to identify effective nonaflatoxigenic strains confirm the importance of strain selection, since not all atoxigenic strains are effective biocontrol agents [52,82,135,140,141,142,143]. Identifying strains that can be used as biocontrol agents is thus a long and complex process.

As shown in Table 3, most studies to date have focused on maize, which is one of the crops most susceptible to aflatoxin contamination. That said, promising trials have also been conducted recently on sorghum in Ghana and sunflower in Tanzania [48].

Some studies evaluated the effectiveness of nonaflatoxigenic strains over multiple years [70,71,82,140,142,144,145,146,147,148,149,150,151], the longest of which was a decade-long study in Nigeria [139]. Multiyear analyses are essential, as shown by Dorner (2009) [152] and Abbas et al. (2006) [148], because the effectiveness of biocontrol agents can vary from year to year, particularly due to fluctuations in the presence of endemic *Aspergillus* species, which are influenced by climatic conditions.

**Table 3:** Inhibitory effect of nonaflatoxigenic *Aspergillus* strains and strain-based biocontrol product on aflatoxin contamination.

Validation Stage	Crop or Matrix	Country	Atoxigenic Strain or Strain-Based Biocontrol Product	Aflatoxin(s) Monitored	Length/Scale of the Study	Reduction in AF Contamination (%)	Reference
In vitro	Culture medium	USA	*A. parasiticus* ATCC 24690; ATCC 24551 AF-1; ATCC 36597 and SRRC 163	AFB1, AFG1	Not reported	83.3 to 92.9%	[153]
In vitro	Culture medium	USA	*A. flavus* Papa 827, 70s-GFP, K49, NRRL 2947, NRRL 29474-20, NRRL 21882, 4-2 and 5-1	AFB1	Not reported	0 to 100%	[131]
In vitro	Culture medium	USA	*A. flavus* SRRC1588	AFB1	Not reported	99.7%	[93]
In vitro	Culture medium	USA	*A. flavus* NRRL 32354 and NRRL 29269	AFB1	Not reported	48.8 to 95.9%	[88]
In vitro	Culture medium	USA	*A. flavus* CA40, LA4-5, VA2-1, GA4-4, LA10-4, MS5-6, NM1-6, SC6-9 and TX9-8	AFB1, AFB2	Not reported	0 to 90%	[154]
In vitro, Grains	Maize	Germany	*A. flavus* AF36	AFB1, AFB2, AFG1, AFG2	Not reported	In vitro: 73 to 100%; Grains: 59 to 100%	[87]
In vitro, Grains, Greenhouse	Maize	Mexico	*A. flavus* CS1	Not reported	Not reported	46.7 to 92.9%	[64]
In vitro, Grains	Rice	Republic of Korea	Atoxigenic *A. flavus* ATCC 9643	AFB1, AFB2	7 days; laboratory assays in triplicate	84%, 95%, and 97% in agar assays	[155]
In vitro, Grains	Peanut seeds	Italy/United Kingdom	Atoxigenic *A. flavus* MUCL 54911 (active ingredient of AF-X1)	AFB1	10 days; laboratory-scale assays	55% in vitro and 71% in peanut seeds	[156]
In vitro, Grains	Maize and peanut	China	Selected indigenous atoxigenic *A. flavus* genotypes from southern China	AFB1	Duration not specified; laboratory scale	74–99%	[157]
In vitro, Grains	Maize and peanut	China	*A. flavus* NRRL 30797	AFB1	Duration not specified; laboratory scale	Up to 90%	[158]
In vitro, Grains, Field	Peanut and field soil	China	Atoxigenic *A. flavus* CA04, SF01, GX06, and GX61	AFB1	7–10 days in vitro; approximately 2.5-month field trial	Field soil: 69.35%; laboratory grains assays reached up to 95%	[159]
Grains	Maize	Argentina	*A. flavus* AS03145, AS04322, AS03802, AS04050, AS05739, AS10202, AS12012, AS04001	AFB1	Not reported	54 to 83%	[143]
Grains and fields	Maize and peanuts	Ghana	*A. flavus* GHM001-5, GHM017-6, GHM109-4, GHM173-6, GHM174-1, GHM287-10, GHM511-3, GHG079-4, GHG083-4, GHG183-7, GHG321-2 and GHG331-8	AFB1, AFB2	50 maize fields, 3 agroecological areas	Grains: 87 to 98% Fields: 50 to 100%	[52]
Grains	Maize	Italy	*A. flavus* endemic isolates and NRRL 21882	AFB1, AFB2, AFG1, AFG2	Not reported	61 to 90%	[69]
Grains	Maize	Kenya	*A. flavus* C6-E, C8-F, E62-L, E63-I, R1-N	AFB1	Not reported	7.1 to 98.3%	[135]
Grains	Maize	Malaysia	*A. flavus* AKR1-, AKR5-, AKL34-, AKL35- and AKL36-	AFB1	Not reported	23 to 79%	[160]
Grains and fields	Maize	Nigeria	*A. flavus* Ab2216, Ak2757, Ak3020, Ak3058, Ka16127, La3108, La3279, La3303, La330, Lo4216, Og0222	AFB1, AFB2	2 years	Grains: 70.1 to 99.9% Fields: 93.4 to 99.3%	[82]
Grains	Maize (N78B-GT)	USA	*A. flavus* AF36	Not reported	Not reported	25 to 73%	[85]
Grains	Maize	Uganda	*A. flavus* and *A. tamarii*	AFB1 (AFG1 and AFG2 in one single sample)	Not reported	50 to 97%	[141]
Grains, Fields	Maize and peanut	China	Atoxigenic *A. flavus* PA51 and PA61	AFB1	Not reported	Maize: 92.13% (PA51), 89.21% (PA61); peanut: 85.69% (PA51), 95.77% (PA61). Field: nearly 80%	[158]
Grains, Greenhouse, Fields	Maize	Italy, USA	*A. flavus* NRRL 30797	Not reported	2 plots of 8ha	70.4 to 85.2%	[65]
Fields	Maize and peanuts	Gambia	*A. flavus* Aflasafe SN01	Not reported	2 years, 40 maize fields	79.8 to 94.8% for maize	[150]
Fields	Maize and groundnut	Tanzania	Aflasafe TZ01 and Aflasafe TZ02 (four native atoxigenic *A. flavus* genotypes per product)	Total aflatoxins	2 years; farmer-field trials in 4 regions	30–92%, depending on crop, region, and season	[161]
Fields	Maize	Mexico	*A. flavus* AF36 Prevail^®^ (VCG YV36)	Total aflatoxins	4 years; 69 field sites	59.0–89.9%	[162]
Fields	Maize	Argentina	*A. flavus* AFCHG2 formulated on a macroporous maize-starch polymer or long-grain rice	AFB1	3 growing seasons; randomized field trials with 3 replicates	Up to 81%	[163]
Fields	Maize	Greece	Selected endemic atoxigenic *A. flavus* strains	Total aflatoxins	3 consecutive field seasons	Complete suppression of aflatoxin accumulation across the three field seasons	[164]
Fields	Groundnut and maize	Mozambique	Aflasafe MWMZ01 (GP5G-8, GP1H-12, MZM594-1, MZM029-7) and Aflasafe MZ02 (GP5G-8, MZG071-6, MZM028-5, MZM250-8)	Total aflatoxins	2 years; 1026 participating farmers across 4 agro-ecological zones	Groundnut: 78–98%; maize: 61–93% (*p* < 0.01)	[165]
Fields	Maize, groundnut, and sorghum	Burkina Faso, Mali, Niger, and Togo	Aflasafe BF01; Aflasafe SN01 in Mali; original and organic Aflasafe BF01 formulations in Togo	Total aflatoxins	2012–2023; 2638 farmer fields	Overall >80% less aflatoxin; crop-, country-, and year-specific results reported separately	[166]
Fields	Maize	Tanzania	Aflasafe TZ01^®^ (four native atoxigenic *A. flavus* strains) (A to Z textile Mills Limited, Arusha, Tanzania)	AFB1, AFB2, AFG1, AFG2	2021–2022 season; 158 fields in 2 districts	AFB1: 62%; no statistically significant reduction was established for the other measured aflatoxins	[167]
Fields	Maize and peanuts	Ghana	*A. flavus* Aflasafe GH01 and GH02	AFB1, AFB2	2 years 400 maize fields (from 0.5 to 2ha), 5 regions	80 to 100% for maize	[140]
Fields	Maize (PR31K18)	Italy	*A. flavus* NRRL 30797 and NRRL 21882	Not reported	2 years, different plots	80 to 96%	[147]
Fields	Maize (Pioneer Hi-Bred PR31K18)	Italy	*A. flavus* NRRL 30797	Not reported	2 years, several plots of 1.5ha	59 to 92%	[145]
Fields	Maize (Pioneer PR33M15)	Italy	*A. flavus* A2085 and A2321	AFB1	3 regions, 2ha plots	83.7 to 94.8%	[70]
Fields	Maize and peanuts	Nigeria	*A. flavus* Ka16127, La3304, La3279 and Og0222	Not reported	10 years	72 to 94%	[139]
Fields	Maize (ACCR-9931-SR)	Nigeria	*A. flavus* La3303, La3304, La3279 and Ka16127	AFB1, AFB2	2 years, 4 different agroecological areas	67 to 95%	[71]
Fields	Peanuts, maize	Senegal	*A. flavus* Ss19-14, MS14-19, M2-7 and M21-11 (=Aflasafe SN01)	AFB1, AFB2, AFG1, AFG2	1 region, 44 plots (from 0.25 to 5ha)	58.3 to 100%	[138]
Fields	Maize (Kerbanis FAO class 500)	Serbia	*A. flavus* Mytoolbox Af01	AFB1	2 years	51 to 83%	[151]
Fields	Maize	Turkey	*A. flavus* NRRL 21882 (=Afla-Guard)	AFB1, AFB2, AFG1, AFG2	2 years, 1 field	98 to 99%	[149]
Fields	Maize	Thailand	*A. flavus* FRR 6023; FRR 6024; FRR 6025 and FRR 6026	Not reported	2 years, 2 different districts, between 3 and 7 plots/experiment	0 to 85%	[142]
Fields	Maize	USA	*A. flavus* NRRL 21882 and Afla-Guard	Not reported	2 years; 64 plots	0 to 76%	[152]
Fields	Maize	USA	*A. flavus* NRRL 30797 (=K49)	Not reported	1 year	65 to 97%	[168]
Fields	Maize, peanuts, cotton	USA	*A. flavus* NRRL 21882, NRRL 21268 and *A. parasiticus* NRRL 21369	AFB1, AFB2, AFG1, AFG2	4 years, 24 plots	66 to 87%	[144]
Fields	Maize (Pioneer 31P42, DKC 67-88, Pioneer 33M52, DK 66-94, DKC 64-69)	USA	*A. flavus* NRRL 21882 (=Afla-Guard); NRRL 18543 (=AF36)	AFB1	2 years, 3 different states	0 to 97.5%	[146]
Fields	Maize (Pioneer 3225 and Pioneer 32R25)	USA	*A. flavus* CT3 and K49	AFB1, AFB2	4 years	43 to 95%	[148]
Fields	Maize (Syngenta N78S)	USA	*A. flavus* NRRL 21882 (=Afla-Guard); NRRL 18543 (=AF36); IC6510; IC6511; IC6512; IC6542	AFB1	1 year	> 70%	[169]
Fields	Maize	USA	*A. flavus* NRRL 21882 (=Afla-Guard), and 30797 (=K49)	AFB1, B2, G1, G2	2 years	0 to 100%	[170]
Fields	Maize (Pioneer Brand 3369A)	USA	*A. flavus*	AFB1	2 years	80 to 95%	[134]
Fields and storage	Maize	Ghana	Aflasafe GH02	AFB1, AFB2, AFG1, AFG2	1 field season plus 6 months of storage; 2 agro-ecological zones	No single overall percentage reported. Mean AFB1: 0.16 vs. 1.02 µg/kg; mean AFG2: 8.55 vs. 11.73 µg/kg (treated vs. untreated); effects varied by toxin, zone, and packaging	[171]

### 3.3. Other Microorganisms Used as Biocontrol Agents

Although numerous studies have investigated the use of microorganisms other than atoxigenic strains to control aflatoxins, the majority were performed in vitro under controlled conditions; greenhouse and field studies do exist, but they remain relatively scarce. As a result, the potential of these microorganisms to serve as effective biocontrol agents against aflatoxins is poorly known. In addition, the studies that do exist focus almost exclusively on maize: only a single study has explored the antifungal effects of bacteria on another cereal (coix seeds, *Coix lacryma-jobi* L.) [172]. The following paragraphs summarize the available studies.

#### 3.3.1. Bacteria

The inhibition of aflatoxin production has been studied in numerous bacterial species belonging to various genera: *Bacillus*, *Lactobacillus*, *Streptomyces*, *Pseudomonas*, *Burkholderia*, *Achromobacter*, *Stenotrophomonas*, *Myroides*, *Nannocystis*, *Pediococcus*, *Serratia*, and *Vibrio* (Table 4). Most of these studies were conducted in vitro. Of the approximately fifty studies reviewed, only three evaluated bacterial effects under greenhouse conditions, and only three in field trials. The bacterial strains studied came from a wide range of sources. Some were isolated directly from crop environments (soil, plant residue, etc.), while others were obtained from marine habitats, preserved foods, animal organs, or fruits. This diversity in bacterial origin may be important when developing a biocontrol agent because it can influence not only the strain’s effectiveness but also its safety profile.

Lactic acid bacteria (LAB) constitute a particularly well-documented group of candidates for bacterial biocontrol. Their antifungal and mycotoxin-mitigation mechanisms, including acidification, antimicrobial metabolite production, competition, toxin adsorption, and enzymatic transformation, were recently reviewed by Mafe and Büsselberg (2026) [46], which served as the main source for discussing the coverage of LAB in this section. To avoid duplicating an already comprehensive synthesis, Table 4 does not provide an exhaustive inventory of primary LAB studies, but includes instead selected studies that were not covered by Mafe and Büsselberg (2026) and that are directly relevant to inhibiting aflatoxigenic fungi or aflatoxin production, together with evidence obtained for other bacterial taxa.

Some studies report highly variable results, further highlighting the need for careful selection of effective strains. For instance, Palumbo, O’keeffe and Abbas (2007) [173] reported that only 443 out of 1018 tested bacterial strains inhibit the growth of *A. flavus*. Nevertheless, several bacterial species from the genera *Bacillus*, *Lactobacillus*, *Pseudomonas*, and *Streptomyces* have been demonstrated to strongly inhibit fungal growth and/or aflatoxin production in in vitro studies.

The genus *Bacillus*, which grows rapidly and is generally considered safe, is the most extensively studied of the bacterial strains [3,174]. Species such as *B. subtilis*, *B. licheniformis*, *B. megaterium*, *B. amyloliquefaciens*, *B. mojavensis*, *B. cereus*, *B. mycoides*, and *B. pumilus* are promising candidates for use as biocontrol agents. However, some of these, such as *B. cereus*, are potentially hazardous and have been associated with outbreaks of foodborne illness (collective food poisoning).

The genus *Pseudomonas* offers the advantage of commonly appearing in soil and being capable of colonizing a variety of ecological niches [175]. Promising results have been obtained with *P. paracarnis*, *P. chlororaphis*, *P. stutzeri*, and *P. fluorescens*. Similarly, *Streptomyces* species such as *S. alboflavus* and *S. roseolus* are commonly found in soil and plant environments and appear effective in regulating aflatoxin production [41]. Other genera and species have also shown promising results, including *Achromobacter*, *Myroides odoratimimus*, *Vibrio gazogenes*, *Serratia marcescens*, and *Burkholderia cepacia*.

The effectiveness of these microorganisms appears to depend strongly on bacterial concentration, substrate, temperature, pH, and water activity [120,176,177,178]. These factors underscore the importance of selecting strains capable of adapting to environmental variability and of conducting field trials before drawing conclusions about the efficacy of a potential biocontrol agent [179,180].

Moreover, some in vitro assays have been performed under conditions that may differ significantly from those encountered in real environments, for example, at pH > 8 and at temperatures exceeding 50 °C [172]. Therefore, although promising in in vitro trials, bacterial strains need to be evaluated under more realistic conditions before they can be considered serious candidates for field application as biocontrol agents. For example, a study on *B. megaterium* showed a marked decrease in biocontrol efficacy upon moving from in vitro conditions to grain-based trials [172]. Field results obtained over multiple years show that certain strains from the *Bacillus*, *Pseudomonas* [181], and *Streptomyces* [182] genera effectively inhibit aflatoxin production, highlighting their potential as biocontrol agents. However, large-scale studies are still needed to confirm these initial findings. Finally, although less extensively studied, other bacterial genera remain promising candidates for use as biocontrol agents.

**Table 4:** Summary of studies of bacteria used to inhibit aflatoxin production and/or the development of aflatoxigenic fungal species.

Bacterial Strain(s)	Validation Stage	Crop or Matrix	Country	Aflatoxigenic Fungal Strain(s) Tested	Observed Effect	Reference
*Achromobacter xylosoxidans NFRI-A1*	In vitro	Culture medium	Japan	*A. parasiticus SYS-4 (NRRL2999)*	No significative impact on fungal growth, total inhibition of AF production	[94]
*Achromobacter*, *Agrobacterium*, *Bacillus*, *Burkholderia*, *Chryseobacterium*, *Pseudomonas*, *Wautersia*, *Stenotrophomonas*, *Variovorax*, *Coryneform bacteria*, *Enteric bacteria* isolated from soil and maize rhizosphere (Mississippi region)	In vitro	Culture medium	USA	*A. flavus*	Reduction in *A. flavus* growth (74 to 89% with *Burkholderia cepacia*, 69 to 96% with *P. fluorescens* et *P. chlororaphis*) Among the 1018 tested strains, 443 inhibited *A. flavus* development	[173]
1018 bacterial isolates representing *Achromobacter*, *Agrobacterium*, *Bacillus*, *Burkholderia*, *Chryseobacterium*, *Pseudomonas*, and other genera	In vitro	Culture medium	United States	*A. flavus*	Of 1018 isolates, 443 inhibited *A. flavus*; *Burkholderia cepacia* reduced growth by 74–89% and *P. fluorescens*/*P. chlororaphis* by 69–96%	[173]
*Bacillus spp.* isolated from fish gut in Amazonia + *B. subtilis ATCC 19659* as a reference	In vitro	Culture medium	Brazil	*A. flavus A12*, *A. sp. A65* and *A. parasiticus 30BL*	Reduction in mycelial development (>60%), sporulation (up to 100%), spore germination % and mycotoxin production (up to 99%)	[104]
*Bacillus sp. Bsp 3/aM*	In vitro, greenhouse, fields (3 years)	Maize	India	*A. flavus UOMMAF1 (KC480445)*	In vitro: inhibition of fungal growth and mycotoxin production Greenhouse: promotion of plant growth Field: reduction in *A. flavus* infection (from 26.2 to 53.5%) and aflatoxin contamination (from 84.2 to 100%)	[181]
*Bacillus strains associated* with Coix seed	In vitro	Coix seed	China	*A. flavus*	*B. velezensis* reduced AFB_1_ to an undetectable or near-undetectable level (approximately 100% inhibition), whereas *B. tequilensis* reduced it by approximately 76%	[183]
*Bacillus amyloliquefaciens A-1*	In vitro, grains	Maize	China	*A. flavus NRRL 335*	In vitro: inhibition of fungal development and dose-dependent inhibition of AFB_1_ production, reaching approximately 99.7% with 20% CFS Grains: inhibition of fungal colonization and approximately 94% reduction in AFB_1_ in maize	[95]
*Bacillus amyloliquefaciens* D-1	In vitro	Coix seed	China	*A. flavus*	VOCs reduced mycelial growth by 71.28%, spore count by 99.2%, and germination by 74.8%, and inhibited aflatoxin biosynthesis	[96]
*Bacillus amyloliquefaciens isolat 1* (*GenBank accession No EU164542*)	In vitro, Greenhouse	Maize	Argentina	*A. flavus MPVPA 2092* and *MPVPA 2094*	In vitro: reduction in *A. flavus* mycelial growth Greenhouse: reduction *A. flavus* population in soil	[179]
*Bacillus amyloliquefaciens* SBCHGBA1	In vitro; food assay	Chilli pepper	India	*A. flavus* MTCC 2798, *A. parasiticus* MTCC 411, and additional *A. flavus* isolates	Completely suppressed aflatoxins in all but one tested *A. flavus* isolate and reduced aflatoxins in chilli pepper by 77%	[184]
*Bacillus cereus B1 and B. thuringiensis K1*	In vitro	Culture medium	Turkey	*A. flavus*	Inhibition of *A. flavus* growth (80–85%), no impact on sporulation and spore germination	[116]
*Bacillus licheniformis BL350-2* isolated from raspberry jam	In vitro, grains	Maize	Qatar	*A. flavus CM5*, *A. parasiticus SB01*	In vitro: inhibition of *A. flavus* CM5 (49%) and *A. parasiticus* SB01 (47%) development, total inhibition of mycotoxin synthesis for most of the tested strains Maize grains: reduction in *A. flavus* CM5 development (88%), inhibition of sporulation and aflatoxins contamination	[98]
*Bacillus megaterium CGMCC 7086* isolated from Yellow Sea	In vitro	Culture medium	China	*A. flavus NRRL 3357*	Reduction in *A. flavus* growth (from 74.67 to 81.15%), reduction in aflatoxins production (>98%)	[185]
*Bacillus megaterium BM344-1* isolated from strawberry jam	In vitro, grains	Maize	Qatar	*A. flavus*	In vitro: inhibition of fungal growth (29.4%), total inhibition of aflatoxins Grains: inhibition of fungal growth (51%), inhibition of aflatoxin synthesis (>70%)	[99]
13 strains of *Bacillus* (*B. mojavensis*, *B. subtilis*, *B. cereus*, *B. megaterium*, *B. mycoides*) isolated from soil and lake sediments	In vitro	Culture medium	Argentina	*A. parasiticus NRRRL 2999*	Reduction in *A. parasiticus* development by 6 out of 13 bacterial strains, reduction in AFB1 production by 97.5% (1 strain)	[100]
*Bacillus paramycoides*	In vitro, grains	Unhulled rice	China	*A. flavus*	VOCs inhibited germination and hyphal growth; in rice, reduced the fungal population by 90.8% and AFB1 by 67%	[101]
*Bacillus pumilus*, 6 isolates	In vitro	Culture medium	USA	*A. parasiticus NRRL 2999*	Reduction in mycelial growth (from 34.4 to 56.4%), reduction in aflatoxin production (from 98.4 to 99.9%)	[186]
*Bacillus safensis* TR-47	In vitro, grains	Peanut	China	*A. flavus*	VOCs significantly inhibited mycelial growth, and conidial germination and completely prevented infection and aflatoxin contamination during storage	[102]
*Bacillus siamensis* 3BS12-4 extracellular compounds	In vitro, grains	Peanut	Thailand	*A. flavus*	Reduced growth by 43.10% in vitro, degraded AFB1 by 96.11%, and inhibited growth on peanut by 97.93%	[103]
*Bacillus subtilis (RCB 3*, *RCB 6*, *RCB 55*, *RCB 90)*; *Pseudomonas solanacearum* (*RCB 5*, *RCB 110*); *Amphibacillus xylanus* (*RCB 27*); *Sporolactobacillus inulinus* (*RCB 93*); *Sporolactobacillus inulinus* (*RCB 196*)	In vitro	Culture medium	Argentina	*A. flavus (RCT 23*, *RCT 22*, *RCT 104*, *RCD 65 et RCI 105)* and *A. parasiticus (RCT 9*, *RCT 20 et RCD 106*)	Inhibition of fungal growth from 0 to 75%; inhibition of aflatoxin accumulation by *B. subtilis* (RCB 6, RCB 55, RCB 90) and *P. solanacearum* RCB 110; Total inhibition of AFB1 by *B. subtilis* RCB 90	[187]
*Bacillus subtilis QST 713*	In vitro	Culture medium	Italy	*A. flavus ITEM 8069*	Total inhibition of fungal growth, inhibition of AFB1 production (from 93.1 to 99.7%)	[106]
*Bacillus subtilis*	In vitro	Culture medium	Iran	*A. parasiticus*	Regulation of fungal growth (up to 92%) and aflatoxin production (up to 100%)	[188]
*Bacillus subtilis QST 713* (*Serenade Max^®^*)(Bayer, Leverkusen, Germany)	Grains	Maize	Greece	*A. flavus A6*,*10*; *D1*,*3*; *12S*	Grains: reduction in sporulation (55.4%), no consistent reduction in AFB_1_ production	[182]
*Bacillus subtilis* RG01	In vitro; grain-storage assay	Rice	China	Toxigenic fungi including *A. flavus*	Suppressed toxigenic fungi and mycotoxin accumulation in stored rice through microbial-community interactions; no single overall percentage was reported	[189]
*Bacillus subtilis NK-330 et NK-C-3* isolated from soil	In vitro, grains	Maize and peanuts	Japan	*A. parasiticus NRRL 2999* and *A. flavus NRRL 3357*	In vitro: complete inhibition (100%) of aflatoxin production Grains: complete inhibition (100%) in peanuts under a 5:1 bacterial-cell-to-fungal-spore ratio; marked inhibition in maize (percentage not reported); fungal growth was markedly inhibited, but not quantified	[190]
*Bacillus subtilis -BSS4*, *B. subtilis BSW*, *P. fluorescens PFMst* et *P. fluorescens PFDL*	In vitro	Culture medium	Iraq	*A. flavus (AFL14)*	Inconsistent regulation of aflatoxin production depending on experimental conditions	[177]
*Bacillus tequilensis (B1) et Bacillus velezensis (B2)* isolated from the rhizosphere of *Camellia sinensis*	In vitro	Culture medium	China	*A. flavus*	Inhibition of fungal development, sporulation and aflatoxin production (AFB1 by 34.22%, AFB2 by 35.22%)	[191]
*Bacillus velezensis HC6* isolated from pig gut	In vitro, grains	Maize	China	*A. parasiticus 3.0124*, *A. flavus LAB08*	In vitro: antagonistic action on *Aspergillus* (from 45.1% to 59.6%) Grains: visible inhibition of fungal growth (not quantified); complete inhibition (100%) of detectable AFB_1_ production with the HC6 cell culture	[108]
*Bacillus velezensis* P1, P7, P11, and P45	In vitro, fruit assay	Grape	Brazil	*A. flavus* and other mycotoxigenic fungi	Grape assay: aflatoxins were not detected in grapes treated with *B. velezensis*, corresponding to complete inhibition (100%) of detectable aflatoxin production	[192]
*Bacillus velezensis* PGB-2	In vitro, seed-storage assay	Peanut, maize, and wheat	China	*A. flavus*	VOCs significantly reduced *A. flavus* infection in all three seed matrices; no single overall percentage was reported	[193]
*Bacillus velezensis* ZH179 and *B. amyloliquefaciens* ZH409/ZH99	In vitro, grains	Maize	China	*A. flavus* HO8-B22	In stored maize, spore inhibition was 59.0–76.3% at 15% moisture and 63.2–66.7% at 20% moisture, depending on strain	[109]
*Burkholderia spp.* Isolated from maize fields	In vitro	Culture medium	Nigeria	*A. flavus*, *A. parasiticus*	Inhibition of fungal growth (48%)	[194]
*Burkholderia contaminans* BC11-1	In vitro	Culture medium	China	*A. flavus*	AFB1 content decreased from 3936 to 135 µg/kg, corresponding to a 96.6% reduction	[195]
Chicken-derived probiotic strains SB1, SB2, and SD2	In vitro, feed assay	Poultry feed	Pakistan	*A. flavus* and other mycotoxigenic fungi	Completely inhibited the tested fungi; reduced spore germination by up to 91.7 ± 0.3% and aflatoxin production by 91.6 ± 1.2%	[196]
Eleven cereal- or grape-derived LAB strains; *Pediococcus pentosaceus* S11sMM and M9MM5b were most effective	In vitro	Culture medium	Spain	*A. flavus*, *A. parasiticus*, *A. carbonarius*, *A. niger*, *A. welwitschiae*, *A. steynii*, and *A. westerdijkiae*	Growth inhibition ranged from 0 to 100%; aflatoxin inhibition ranged from 19 to 60.8%, depending on strain and conditions	[178]
*Flavobacterium johnsoniae* TR-18	In vitro, grains	Peanut	China	*A. flavus*	Peanut storage assay: complete inhibition (100%) of detectable AFB_1_ and AFB_2_ production	[110]
*Klebsiella aerogenes* KTTM	In vitro, grains	Peanut	Japan	*A. flavus*	Strong inhibition of AF production without affecting fungal growth; approximately 95–100% reduction in total AFB_1_ + AFB_2_ was observed	[111]
*Lacticaseibacillus paracasei* F50	In vitro, grains	Maize	China	*A. flavus*	Reduced fungal growth and aflatoxin contamination in maize; efficacy depended on treatment and matrix conditions, with no single overall percentage reported	[197]
*Nannocystis exedens ATCC 25963*	In vitro	Culture medium	USA	*A. flavus ATCC 16875*, *ATCC 26946* and *A. parasiticus NRRL 3145*	Reduction in *A. flavus* and *A. parasiticus* development (from 50 to 59%)	[198]
*Paenibacillus polymixa CECT 155* isolated from soil and decaying plants	In vitro	Culture medium	Spain	*A. flavus ITEM 8111*	Reduction in AFB1 production (55%)	[199]
*Pantoea agglomerans* B-379N (best-performing among tested bacterial and yeast isolates)	Field	Maize	Greece	Natural infection by aflatoxin- and fumonisin-producing fungi	Consistently showed the highest efficacy and reduced aflatoxins and fumonisins under natural infection; no single overall percentage was reported	[200]
*Pediococcus pentosaceus* (four strains), *Weissella paramesenteroides* (two strains), and *Lactobacillus crustorum* RL48-10	In vitro	Culture medium	Republic of Korea	*A. flavus*	Reduced AFB_1_ concentration through species-dependent combinations of production inhibition and cell binding; approximately 10–90% reduction depending on strain and treatment fraction	[201]
*Pseudomonas sp.* 20EI1	In vitro	Culture medium	United States	*A. flavus*	In vitro: inhibition of fungal growth by 84.6%, decreasing to 45.9% with iron supplementation; inhibition of AFB_1_ production by 99.8% without iron and 99.7% with iron; cyclopiazonic acid and kojic acid production were reduced by 97.6% and 99.9%, respectively	[202]
*Pseudomonas fluorescens PB27*	In vitro	Culture medium	Turkey	*A. flavus*	Inhibition of *A. flavus* development (70%), no impact on sporulation, reduction in germination by 20%	[116]
*Pseudomonas putida Has 1/c*	In vitro, greenhouse, field (3 years)	Maize	India	*A. flavus UOMMAF1 (KC480445)*	In vitro: strongest antagonistic activity against *A. flavus* among the bacterial isolates (qualitative score; percentage not reported); reduction in aflatoxin production (percentage not reported). Greenhouse: promotion of plant growth Field: significant reduction in *A. flavus* infection (from 41.9 to 48.8%) and aflatoxin contamination (from 21.1 to 50%)	[181]
*Pseudomonas stutzeri YM6* isolated from marine sediments	In vitro, grains	Maize and peanuts	China	*A. flavus*	In vitro: inhibition of fungal growth (up to 100%) and spore germination Grains: inhibition of fungal infection and aflatoxin production (100%)	[117]
*Pseudomonas. paracarnis Ps-4*, *P. chlororaphis Ps-66* and *Ps-68* isolated from maize rhizosphere	In vitro	Culture medium	Hungary	*A. flavus*	No significant effect on fungal growth; >99% inhibition of AFB_1_ production on solid medium and complete inhibition (100%) in liquid culture	[175]
*Serratia marcescens JPP1* isolated from peanut shell	In vitro	Culture medium	China	*A. parasiticus*	Inhibition of mycelial growth (>95%) and aflatoxin production (>98%)	[118]
*Stenotrophomonas rhizophila* isolated from soil	In vitro	Culture medium	Japan	*A. parasiticus ATCC 24690* and *NRRL 2999*, *A. flavus IFM 47798*	No significant effect on fungal growth; dose-dependent inhibition of aflatoxin production, reaching 100% for *A. flavus* IFM 47798; percentage not reported for *A. parasiticus* NRRL 2999	[120]
*Streptomyces alboflavus TD-1* isolated from soil	In vitro	Culture medium	China	*A. flavus*	Inhibition of mycelial growth and conidial germination up to 100%, and inhibition of AFB_1_ production by 93.68%; deformation of hyphae and conidia and alteration of mitochondrial function	[121]
*Streptomyces griseoviridis (Mycostop^®^)*	Grains, Field (2 years)	Maize	Greece	*A. flavus A6*,*10*; *D1*,*3*; *12S*	Grains: reduction in sporulation (48.2%) and significant reduction in AFB_1_ production (percentage not reported). Field: reduction in fungal infection (16.5%) and AFB1 contamination (43.05%)	[182]
*Streptomyces roseolus*	In vitro	Culture medium	France	*A. flavus NRRL 62477*	Reduction in AFB1 (almost 100%) and Kojic acid (37%) synthesis; no significant effect on cyclopiazonic acid production	[203]
*Streptomyces roseolus*	In vitro	Culture medium	France	*A. flavus NRRL 62477*	Inhibition of fungal growth (77%) and AFB1 production (almost 100%)	[204]
*Streptomyces sp. TD-1* identified as *Streptomyces alboflavus*, isolated from soil	In vitro	Culture medium	China	*A. flavus*	Inhibition of fungal development (>24.8%)	[205]
37 isolates of *Streptomyces sp.* isolated from soil	In vitro	Culture medium	France	*A. flavus NRRL 62477*	Frequent lack of impact on fungal growth, reduction in AFB1 concentration (from 4.4 to 38.6% by 27 out of the 37 tested isolates)	[206]
*Vibrio gazogenes ATCC 43942*	In vitro, grains	Maize	USA	*A. flavus NRRL 3357 et AF70*	In vitro: inhibition of aflatoxin synthesis (from 50 to 100% depending on bacterial concentration) Grains: reduction in aflatoxins production (>90%), of hyphae fusion (>98%)	[122]

#### 3.3.2. Micromycetes

Multiple filamentous fungal species have been investigated for their capacity to suppress the growth of *Aspergillus* section *Flavi* and to reduce aflatoxin production in cereal grains (Table 5).

Several species of the genus *Trichoderma* have shown a strong potential to inhibit aflatoxin production in in vitro studies [61,207,208] as well as on maize grains [209]. These results are supported by greenhouse and field trials. For example, Sivparsad and Laing (2016) [210] and Aiyaz et al. (2015) [181] reported a reduction in maize-ear infection by *A. flavus* and a decrease in aflatoxin production following both the spraying of a *Trichoderma harzianum* strain [210] and the application of talc-based *T. asperellum* formulations on maize plants [181]. Conversely, Lagogianni and Tsitsigiannis (2019) [182] reported no significant effect of *T. harzianum* under field conditions. Nevertheless, convincing results, particularly those of Aiyaz et al. (2015) [181], obtained over three consecutive years in the field, indicate that certain *Trichoderma* species are promising as biocontrol agents. However, larger-scale research is required before potential commercialization.

Other fungal species have also been investigated. Strains of the genus *Aspergillus* yielded promising results in both in vitro and greenhouse experiments [211,212]. Geisen (2000) [213] reported that a *Penicillium* species inhibits secondary metabolite production by *A. flavus*, although the overall antifungal effect is limited. Additional fungi of interest include *Dichotomopilus erectus* [194], *Rhizopus oryzae* [212], and the basidiomycete *Trametes versicolor* through its polysaccharide Tramesan [214]. The latter has the advantage of being synthetically producible, facilitating its potential for large-scale development.

Voloshchuk et al. (2024) and Geisen (2000) [61,213] showed that fluctuations in temperature, humidity, and nutrient availability can significantly impact the efficacy of fungi as biocontrol agents. In addition, Lagogianni and Tsitsigiannis (2019) [182] reported notable differences between grain-based assays and field trials. These findings underscore the importance of considering future environmental conditions during early in vitro testing and the necessity of conducting trials under real-world conditions to properly assess the potential of fungal species as biocontrol agents.

**Table 5:** Micromycetes used to inhibit aflatoxin production and/or the development of aflatoxigenic fungi.

Species	Validation Stage (In Vitro, Grain, Greenhouse, Field)	Crop	Country	Aflatoxigenic Species	Nature of the Effect	Reference
*Alternaria alstroemeriae F1*	In vitro	/	China	*A. flavus* (peanut isolate)	Growth inhibition was 54.91 ± 0.55%; AFB1 and AFB2 decreased significantly, with downregulation of 14 positive regulators of aflatoxin biosynthesis (toxin inhibition was not reported as a percentage)	[215]
*Nigrograna chlamydospora B143*	In vitro	/	China	*A. flavus GP15-4*	Plate confrontation inhibited growth by 44.16 ± 6.14%; a petroleum-ether crude extract (25 mg/mL) inhibited mycelial growth by 70.84 ± 4.29%, spore germination by 98.23 ± 0.10%, and AFB1 production by 35.88%	[216]
*Trichoderma asperrellum*; *T. atroviridae*; *T. citrinoviride*; *T. harzianum*; *T. inhamatum*; *T. parceramosus*; *T. polysporum*; *T. viride*	In vitro	/	China	*A. flavus ITEM 9 (=NRRL 3251)*	Reduction in *A. flavus* development from 0 to 39.7%; inhibition of AFB1 production up to 84.9%	[207]
*Trametes versicolor*	In vitro	/	Croatia	*A. flavus NRRL 3357*	No effect on fungal growth, reduction in AFB1 production up to 90%	[214]
*Penicillium nalgiovense BFE66*, *BFE67 et BFE474*	In vitro	/	Germany	*A. flavus BFE96*	Inhibition of *A. flavus* development	[213]
*Trichoderma harzianum*; *Dichotomopilus erectus*	In vitro	/	Nigeria	*A. flavus*, *A. parasiticus*	Mean reduction in *Aspergillus* growth of 74% for *T. harzianum* (but only 30% for *A. flavus*) and 50% for *D. erectus*	[194]
*Fusarium moniliforme*; *Mucor hiemalis*; *Trichoderma sp.*; *T. harzianum*; *T. viride*	In vitro	/	Saudi Arabia	*A. flavus*	Reduction in mycelial development (74 to 90%) with *T. viride*, *Mucor. hiemalis*, *T. harzianum*, *T. sp. et F. moniliforme* and reduction in AFB1 production (78 to 91%) with *T. harzianum*, *T. viride*, *T. sp et F. moniliforme*	[126]
*Trichoderma viride* and *T. harzianum*	In vitro	/	South Africa	*A. flavus*	Reduction in *A. flavus* development	[208]
*Trichoderma asperellum OSK-38* and *BTU*; *T. atroviride PNB12-IAI*; *T. harzianum OSK-21* and *OSK-34*; *T. virens OSK-13* and *OSK-36*; *T. viride OSK-22.*	In vitro	/	USA	*A. parasiticus B62*, *A. flavus NRRL 3357*	Reduction in *A. parasiticus* (38–42%) and *A. flavus* (40–45%) growth AFB1 inhibition was assessed by TLC (not quantified)	[61]
*Aspergillus fumigatus* and *A. terreus*	In vitro, greenhouse	Maize	Egypt	*A. flavus*	In vitro: reduction in *A. flavus* growth (77.6% with *A. fumigatus* and 39.7% with *A. terreus*) and aflatoxin production: AFB1 (90.9%), AFB2 (93.1%) Greenhouse: reduction in aflatoxin production: AFB1 (41.74%), AFB2 (62.70%)	[211]
*Trichoderma harzianum T77* (Biocontrol agent registered as *Eco-77*)	In vitro, greenhouse, field (1 year)	Maize	South Africa	*A. flavus PPRI 1314*	In vitro: inhibition of *A. flavus* growth was observed but not quantified Greenhouse and field: T77 applied at 10 and 12 days post-midsilk significantly reduced kernel infection and aflatoxin contamination (exact percentages not reported)	[210]
*Trichoderma harzianum*; *T. asperellum*	In vitro, grains, greenhouse	Maize	Pakistan	*A. flavus*; *A. parasiticus*	Co-culture with *T. harzianum* reduced total aflatoxins by 99.3%; in maize inoculated with *A. flavus*, aflatoxins decreased from 5120 ± 560 µg/kg to 50.1 ± 1.10 µg/kg with *T. harzianum* and 63.1 ± 3.1 µg/kg with *T. asperellum*	[217]
*Trichoderma asperelloides* SKRU-01	In vitro, grains	Peanuts	Thailand	*A. parasiticus* TISTR 3276; *A. flavus* PSRDC-4	VOCs inhibited hyphal growth by 75.67% and 71.33%, and spore germination by 70.88% and 67.41%, respectively; acetophenone (MIC: 1.0 µL/mL in solid medium or 1.0 µL/L in vapor) completely inhibited both strains In peanuts, VOCs reduced mold density and AFB1 production (percentage not reported)	[218]
*T. asperellum M-5* and *T-2*	In vitro, greenhouse, field (3 years)	Maize	India	*A. flavus UOMMAF1 (KC480445)*	In vitro: reduction in *A. flavus* growth from 61.3 to 80.3% with *T. asperellum* strains Greenhouse: improvement of plant development Field: reduction of *A. flavus* infection (53.5 to 64.3% for T-2 strain and -7% to 44.2% for M-5 strain) Reduction in aflatoxin contamination ranging from 47.4 to 100% for T-2 strain and from 36.8 to 84% for M-5 strain	[181]
*Trichoderma harzianum (Trianum^®^)*	Grains, field (2 years)	Maize	Greece	*A. flavus A6*,*10*	Grains: no impact on fungal growth, AFB1 biosynthesis was reduced qualitatively by TLC (not quantified) Field: reduction in fungal infection, but AFB1 reduction was not statistically significant	[182]
*Trichoderma harzianum* K179	In vitro, field (2 years)	Maize	Serbia	*A. flavus K328*	In vitro: maximum inhibition-zone diameter was 36.67 mm Field (2022): AFB1 and AFB2 were not detected (<0.4 µg/kg) in *T. harzianum* treatments versus 2.9–3.2 and 0.6–0.8 µg/kg, respectively, in untreated controls	[219]

#### 3.3.3. Yeasts

The potential of several yeast genera, including *Candida*, *Saccharomyces*, *Cyberlindnera*, *Debaryomyces*, *Hanseniaspora*, *Kluyveromyces*, *Cryptococcus*, *Rhodotorula*, and *Wickerhamomyces*, to counteract aflatoxins has also been investigated (see the results in Table 6).

The yeasts presented in Table 6 originate from various sources, including cheeses [220,221], rice leaves, sugarcane, maize [222], nuts [223], and marine environments [224]. In most studies, certain yeast strains revealed a strong capacity to inhibit aflatoxin production, although the results vary. Most of these studies were conducted in vitro. Some of them tested yeasts under different conditions (water activity, pH, temperature, oxygen availability, etc.) [179,225,226,227]. Nonetheless, greenhouse trials [179] and field trials, such as for the yeast *Aureobasidium pullulans* [182], are essential to confirm the potential use of these microorganisms on crops.

**Table 6:** Use of yeasts to modulate aflatoxin production and/or the development of aflatoxigenic fungi.

Species	Validation Stage	Crop	Country	Aflatoxigenic Species	Observed Effect	Reference
*Aureobasidium melanogenum DAP1*	In vitro, grains	Nutmeg	Indonesia	*A. flavus*	Dual-culture growth inhibition was 43.6%; the separately reported 50.4% decrease in AFB1 was a degradation endpoint and is not treated here as inhibition of biosynthesis	[228]
*Aureobasidium pullulans* (*Botector^®^:* activity demonstrated against *Botrytis cinerea* in vines) (De sangosse, Agen, France)	Grains, Field (2 years, 30 plants/experiment)	Maize	Greece	*A. flavus A6*,*10*, *D1*,*3*, *12S*	Both on grains and in the field: suppression of symptoms of *A. flavus* infection and aflatoxin contamination	[182]
*Candida chrysomelidarum LCA 75*; *Rhodotorula toruloides LCA 102*	In vitro	/	Indonesia	*A. flavus P8 (UNJCC)*	Dual culture inhibited mycelial growth by 32.32% (LCA 75) and 32.30% (LCA 102); aflatoxin production was not quantified	[229]
*Candida nivariensis DMKU-CE18* isolated from rice leaves	In vitro, grains	Maize	Thailand	*A. flavus A39*	In vitro: inhibition of mycelial growth (64.9%) and conidia germination (49.3%) Grains: reduction in aflatoxin contamination (74.8%)	[222]
*Candida parapsilosis IP1698*	In vitro	/	Iran	*A. flavus*, *A. parasiticus ATCC15517*	No impact on fungal development, inhibition of aflatoxin synthesis (92.98% to 99.59%)	[225]
*Cyberlindnera jadinii 273*	In vitro	/	Qatar	*A. flavus*	Inhibition of fungal development	[230]
*Debaryomyces hansenii BCS003*	In vitro, grains	Maize	Mexico	*Aspergillus sp.*	In vitro: inhibition of mycelial growth (97.2 to 98.3%) Grains: partial inhibition of mycelial development, retardation of fungal infection (up to 24h)	[224]
*Geotrichum candidum XG1*	In vitro, grains	Maize	China	*A. flavus*	During 75-day corn storage, XG1 reduced fungal and aflatoxin contamination by >90%; 2-phenylethanol was identified as a key antifungal volatile	[231]
*Hanseniaspora opuntiae L479* and *Hanseniasporauvarum L793*	In vitro	/	Spain	*A. flavus CQ8*	Inhibition of fungal growth by 17.10% (L479, day 10) and 34.43% (L793, day 3); at day 3, AFB1 decreased from 451 ± 621 µg/kg (control) to 47 ± 11 µg/kg with L479 (89.6%) and 3 ± 3 µg/kg with L793 (99.3%)	[232]
*Hanseniaspora uvarum L793*; *Metschnikowia pulcherrima L672*	In vitro (dry-fig agar)	Dried figs	Spain and Italy	*A. flavus M114*	After 8 days, mycelial biomass was reduced by 94.7% (L672) and 94.1% (L793); L672 reduced AFB1 by 94.0% and AFB2 by 88.4%; L793 completely inhibited all detected aflatoxins	[233]
*Kluyveromyces L14 et L16*	In vitro, greenhouse	Maize	Argentina	*A. flavus MPVPA 2092 et MPVPA 2094*	In vitro: reduction in fungal growth at water activities of 0.99, 0.97, 0.95, and 0.93 Greenhouse: reduction in *A. flavus* population in the soil inoculated with *Kluyveromyces sp.* L16	[179]
*Kluyveromyces lactis*	In vitro	/	Iran	*A. parasiticus*	Inhibition of *A. parasiticus* growth and aflatoxin production (97.9%)	[234]
*Kluyveromyces spp Y2*, *Y9*, *Y11*, *Y14*, *Y16*, *Y21*, *Y22*, *Y25*, *Y30*, *Y33 et Y36*	In vitro	/	Argentina	*A. flavus HD 65*, *HI 105*, *HT 22*, *HT 23* and *HT 104*; *A. parasiticus HD 106*, *HT 9* and *HT 20)*	Inhibition of fungal growth (from 0 to 100% depending on water activity) and of aflatoxin production for the majority of tested strains	[226]
*Meyerozyma caribbica Y11A*; *Saccharomyces/Meyerozyma isolates Y18A*, *Y35A*, *Y61A and Y70A*	In vitro	Maize	Peru	*A. flavus Af4*	Co-inoculation inhibited growth by up to 89%; VOCs from the five selected isolates inhibited growth by 49–86%; total aflatoxins were reduced by 98% (Y11A), 93% (Y18A), 76% (Y35A), and 41% (Y61A); Y70A did not inhibit aflatoxins	[235]
*Pseudozyma hubeiensis BUP*	In vitro	Nutmeg	Indonesia	*A. flavus*	Dual-culture growth inhibition was 46.1%; the separately reported 53.03% decrease in preformed AFB1 was a decontamination endpoint and is not treated here as inhibition of aflatoxin biosynthesis	[236]
*Pseudozyma hubeiensis DP 1341a* and *DP 1342*	In vitro	Nutmeg	Indonesia	*A. flavus BIO 33210*	Dual-culture growth inhibition was 47.25 ± 1.66% and 55.98 ± 1.31%, respectively; VOC-mediated inhibition was 32.79 ± 1.01% and 54.33 ± 3.13%, respectively; aflatoxin production was not quantified	[237]
*Saccharomyces cerevisiae*	In vitro	Broiler-chicken rations	Egypt	*Aflatoxigenic A. flavus (OP698331.1*; *OP698332.1)*	Growth of all tested aflatoxigenic isolates was inhibited; the inhibition zone was reported as 24.6 ± 0.64 mm^2^; aflatoxin production under yeast treatment was not quantified	[238]
*Saccharomyces cerevisiae AUMC13706 (MZ889111)*	In vitro, food model	Minced meat	Egypt	Five aflatoxigenic *A. flavus* isolates	Growth inhibition on YMA was 3.84–22.22%, and total aflatoxin production on YES was reduced by 34.55–90.41%; in chilled minced meat, effects on growth and aflatoxigenicity were not significant; added-toxin detoxification was excluded	[239]
*Saccharomyces cerevisiae EBF 101*, *PGUP102*, *HSFI103*, *MARH104*, *116*, *117*, *120*, *122* isolated from cheeses or baker yeasts	In vitro	/	Egypt	*A. flavus Z103*	Reduction in fungal growth (>74%), reduction in AFB1 production (99.8%)	[221]
*Saccharomyces cerevisiae* isolated from local soil	In vitro	/	India	*A. flavus*	Inhibition of mycelial development (up to 92.1%) and AFB1 production (from 52.19 to 99.01%)	[240]
*Saccharomyces cerevisiae RC008* and *RC016*	In vitro	/	Argentina	*A. parasiticus NRRL2999*	Inhibition fungal growth and AFB1 production (from 81 to 98% depending on the strains and experimental conditions)	[227]
*Wickerhamomyces anomalus WRL-076*	In vitro	/	Albania	*A. flavus CA28*, *CA42*, *CA90* and *M52*	Reduction in fungal biomass (up to 60%), conidia number (>95%), and aflatoxin concentration (almost total inhibition)	[241]
*Williopsis saturnus var. saturnus with an EPS film coating*	Grains (storage)	Red pepper	Türkiye	*A. flavus MAM-200736*	Versus the uncoated inoculated control, the yeast–EPS coating reduced AFB1/total AF by 49.3%/51.3% at day 6 and 32.9%/26.6% at day 10	[242]
Yeast isolated from almonds, pistachios, nuts: *Candida guilliermondii WRL-015*, *Cryptococcus laurentii WRL-024*, *Candida krusei WRL-038*, *Rhodotorula mucilaginosa WRL-053*, *Pichia anomala WRL-076* and *Candida oleophila WRL-084*,	In vitro	/	USA	*A. flavus Papa 827*, *A. flavus 42-12 (NRRL-25347)*	Total inhibition of fungal growth and aflatoxin production	[223]

Most of the studies primarily aimed to verify that yeasts may be used for post-harvest contamination control. However, in-field application may also be feasible and should be further explored.

### 3.4. Biosafety and Environmental Risks Associated with Live Microorganisms as Biocontrol Agents

Although the use of microorganisms as aflatoxin biocontrol agents is generally considered a promising and sustainable strategy, several risks and concerns have also been identified. These concerns do not apply equally to all microbial biocontrol agents. The potential production of other mycotoxins is primarily associated with nonaflatoxigenic fungal strains, whereas genetic stability and environmental safety should be considered for all live microbial biocontrol agents, including fungi and bacteria. A careful, science-based assessment of these risks is essential to ensure the long-term safety and effectiveness of biocontrol products. 

#### 3.4.1. Production of Secondary Metabolites

The selection of atoxigenic strains was initially based solely on the absence of aflatoxin production. However, the inability to produce aflatoxins does not mean that nonaflatoxigenic strains are incapable of producing other mycotoxins [81,84]. Uka et al. (2019) [243] examined the ability of more than 50 *A. flavus* strains to produce 50 toxic secondary metabolites and reported that all strains produced at least one toxin. In the same study, the tested commercial biocontrol strains (AF36 or NRRL 21882) produced 11 toxic secondary metabolites. 

For example, the widely used AF36 strain produces cyclopiazonic acid (CPA), a toxic secondary metabolite produced under the same environmental conditions as AFB1, as well as other CPA-like compounds, kojic acid, and aflatrem. The NRRL 21882 strain produces several CPA-like compounds and expresses the genes associated with kojic acid and aflatrem production [84,244,245]. On the other hand, strain K49 cannot produce this mycotoxin because it carries a point mutation in the *pks-nrps* gene of the CPA biosynthetic cluster [246]. However, its potential to produce other mycotoxins has yet to be explored [84]. The term “atoxigenic” is used to describe certain strains and is therefore often misleading; only the term “nonaflatoxigenic” is generally justified [84]. Although the mycotoxins other than aflatoxins produced by these strains currently appear to be less toxic, the risk they may represent—particularly in the case of synergistic effects—should not be overlooked. It is therefore essential to consider the presence of these other secondary metabolites when selecting and using these fungi as future biocontrol agents to ensure that they truly improve the sanitary quality of harvested crops [143].

#### 3.4.2. Genetic Stability and Evolutionary Risks

Microorganisms, including those used as biocontrol agents, are dynamic organisms capable of adaptation and evolution. The genetic stability of these strains may therefore raise concerns. Long-term studies of fungal communities indicate that clonal reproduction, accompanied by spontaneous mutation, is the predominant reproductive mode. Sexual recombination appears to remain highly limited in nature [50,51,54,247,248]. For instance, Atehnkeng et al. (2008) [82] showed that, after 20 cycles, none of the initially nonaflatoxigenic isolates began producing aflatoxins.

However, sexual reproduction, although rare, must be considered, because ignoring it would hinder the development of this biocontrol strategy. Under real-world conditions, sexual reproduction can be favored due to environmental stress, particularly when fungal strains are geographically displaced by wind or insects [84,249,250]. Genetic exchanges may occur between toxigenic and atoxigenic strains, potentially modifying the biocontrol agents, which could then become pathogenic. Horn, Moore, and Carbone (2009) [251] used intraspecific mating experiments in the laboratory to prove that sexual reproduction is possible in *A. flavus*. Currently, nonaflatoxigenic strains are selected without considering fertility, and most studies have no information on the mating type of the strains used. Knowledge of this parameter would allow for even greater stability of the biocontrol agent in the natural environment [84]. Thus, rendering nonfunctional the *MAT* gene of one or more of the nonaflatoxigenic strains would increase the stability of the biocontrol agent [84].

In addition, genetic recombination (parasexuality) can occur between strains (mostly clonal) belonging to the same VCG [252,253,254]. Its frequency under natural conditions is unknown. Parasexuality could allow intrinsically identical fungi to adapt and evolve more rapidly than through random mutations—for example, by allowing them to regain the ability to produce aflatoxins [255]. Therefore, a safer approach would be to use atoxigenic strains carrying major mutations (such as the absence of a gene cluster) rather than strains with only a single defective gene within a functional cluster [84]. 

#### 3.4.3. Biosafety, Environmental Safety, and Nontarget Effects

The introduction into agroecosystems of fungal or bacterial microorganisms raises legitimate concerns about possible ecological effects. Although using native or locally adapted strains may reduce this risk [256], a comprehensive environmental assessment should consider not only changes in the composition of microbial communities but also possible effects on beneficial microorganisms, insects, soil fauna, host-plant health, and ecosystem functions. For example, the biocontrol product Afla-Guard (*A. flavus* NRRL 21882) reduces not only *A. flavus* contamination but also the presence of *Fusarium verticillioides*, which may affect fumonisin levels [257]. Although the absence of *Fusarium verticillioides* may offer certain benefits, its broader ecological impact, particularly on other microbial populations, remains uncertain.

For bacterial biocontrol agents, studies such as those by Pereira, Nesci, and Etcheverry (2007; 2009) [258,259] show in field trials that strains such as *B. amyloliquefaciens* and *Microbacterium oleovorans* do not significantly alter rhizospheric or endophytic microbial diversity. Although these findings provide useful strain-specific evidence, they cannot be generalized to other microbial agents, formulations, crops, or environments. Data concerning long-term nontarget effects resulting from repeated field application remain limited.

Environmental compatibility alone is insufficient to establish the overall biosafety of a microbial biocontrol product. Safety should be assessed at both the strain and formulated-product levels and should not be inferred solely from species identity or natural occurrence. Candidate microbial agents should therefore be evaluated, where relevant, for undesirable secondary metabolites, virulence traits, clinically relevant antimicrobial-resistance determinants, pathogenicity, allergenicity, and persistence. For food and feed applications, candidate microbial agents should be evaluated for viable cells or spores, secreted metabolites, and formulation components remaining in harvested commodities to ensure that aflatoxin mitigation does not introduce other hazards [81,84,231].

#### 3.4.4. Resistance Development and Long-Term Efficacy

To date, insufficient research has focused on the development of resistance to aflatoxin biocontrol agents. Nevertheless, repeated application may produce selection pressure on toxigenic fungal populations or alter their competitive structure, potentially favoring genotypes that are less sensitive to antagonistic interactions or antimicrobial metabolites. Although the multiple mechanisms deployed by live biocontrol agents may reduce this risk compared with single-site chemical treatments, they do not eliminate it. Long-term surveillance should therefore monitor changes in fungal population composition, toxigenicity, sensitivity to the biocontrol agent, and field efficacy. Integration with GAP and, where technically justified, the rotation or combination of agents with different mechanisms of action may further reduce selection pressure [50,51,54,84,247,248,256].

## 4. Use of Plant-Based Substances as Biocontrol Agents

In addition to atoxigenic strains and the microorganisms described above, the use of natural plant-derived compounds is a promising strategy to combat both mycotoxins and associated pathogenic fungi as part of an integrated pre- and post-harvest management strategy [260].

Plants produce secondary metabolites from extremely diverse chemical families and partake in a wide range of biological activities, including antioxidant, antimicrobial, antifungal, and insecticidal activities [3,261,262,263,264,265]. These compounds can be involved in plant growth or in defense mechanisms against insects, bacteria, and fungi [3,266]. Natural molecules and extracts that produce fungicidal or antimycotoxin effects can be grouped into three categories: essential oils, plant extracts, and isolated compounds [3,260].

### 4.1. Mechanisms of Action

Plant-derived substances, with their complex chemical composition and diverse modes of action, have significant potential for controlling aflatoxin contamination. Depending on the extract or compound involved, aflatoxin accumulation may be reduced by inhibition of fungal growth, specific inhibition of aflatoxin biosynthesis, or a combination of both.

#### 4.1.1. Reduction in Aflatoxin Accumulation Through Fungal Growth Inhibition

Plant-derived substances can indirectly reduce aflatoxin accumulation by inhibiting spore germination, mycelial growth, or fungal survival. This indirect mode of action reduces toxin accumulation by suppressing fungal development and should not be interpreted as evidence of a specific effect on the aflatoxin biosynthetic pathway. The magnitude of this antifungal activity depends on the compound or extract and its concentration.

One of the primary mechanisms of essential oils involves direct inhibition of fungal development through disruption of cell membranes and organelle function. Lipophilic compounds such as eugenol, thymol, and citral present in essential oils or as isolated pure molecules compromise the integrity of fungal membranes, which increases permeability, thereby causing loss of cellular content and eventual cell death [267,268,269]. This action is often linked to the hydrophobic nature of these compounds, which enables their interaction with lipid bilayers.

Regarding phenolic compounds such as eugenol, thymol, and carvacrol, fungal-growth inhibition may be due to

(i) Disruption of the plasma membrane, resulting in increased permeability, dissipation of the pH gradient and membrane potential, leakage of ATP and other cellular constituents, and inhibition of membrane-associated enzymes. These effects can impair spore germination, germ-tube elongation, and mycelial development [270,271].
(ii) Interaction with membrane proteins, altering their conformation and functionality and contributing to the accumulation of reactive oxygen species (ROS) [272].

In plant materials containing glucosinolates, antifungal activity is mainly attributed to their breakdown products, particularly isothiocyanates. Their proposed mechanisms include

(i) Inhibition of fungal oxygen uptake due to uncoupling of oxidative phosphorylation in fungal mitochondria, which blocks the coupling between electron transport and phosphorylation reactions, thus impairing ATP synthesis [273].
(ii) Formation of ROS, causing intolerable oxidative stress in fungal cells and irreparable damage to fungal macromolecules [274].
(iii) Nonspecific and irreversible interaction of isothiocyanates with sulfhydryl groups, disulfide bonds, and amino groups of protein and amino acid residues [273,275].
(iv) Interaction of certain isothiocyanates, such as butenyl-isothiocyanate, with enzymes in the plasma membrane, leading to fungal-growth inhibition or cell death [276].

Together, these mechanisms suppress fungal development and consequently reduce the capacity of aflatoxigenic fungi to accumulate aflatoxins.

#### 4.1.2. Specific Inhibition of Aflatoxin Biosynthesis

Some plant-derived substances can notably reduce aflatoxin production without substantially affecting fungal growth, indicative of a specific effect on toxin biosynthesis rather than an indirect consequence of growth inhibition. At the molecular level, the reported responses include

(i) Downregulation of the pathway-specific regulatory genes *aflR* and *aflS*, which coordinate the expression of the aflatoxin biosynthetic gene cluster [277].
(ii) Repression of structural genes that encode enzymes involved in various stages of aflatoxin biosynthesis [277,278].
(iii) Modulation of global regulators of secondary metabolism and genes involved in the oxidative stress response, reflecting the close relationship between fungal redox status and aflatoxin production [1].

A well-documented example of such a plant-derived substance is the aqueous extract of *Micromeria graeca*. At 10 mg/mL, this extract reduces AFB1 production by 99.2% without inhibiting fungal growth. This effect is associated with an approximately threefold downregulation of *aflR* and *aflS*, extensive repression of the AFB1 biosynthetic gene cluster, and modulation of global regulators and genes involved in the oxidative stress response [277].

Similarly, an aqueous extract of *Mimosa tenuiflora* stem bark reduces AFB1 production by 50% and 66% at 0.15 and 0.20 mg dry matter/mL, respectively, while fungal growth is reduced by no more than 3%. The treatment downregulates *aflR*, *aflS*, and structural genes involved in the early, intermediate, and late stages of AFB1 biosynthesis. It also modulates several genes associated with global regulation and the oxidative stress response, including *mtfA*, *atfA*, *msnA*, *catA*, and *sod1*. Bioguided fractionation further supports a major contribution of condensed tannins to this anti-AFB1 activity [279].

Together, these results show that plant-derived substances can suppress specific aflatoxin biosynthesis routes by making coordinated transcriptional changes that affect both aflatoxin gene cluster expression and broader regulatory networks.

### 4.2. Essential Oils

Essential oils are obtained from plant raw materials and are mixtures of volatile secondary metabolites, some of which belong to the terpene family. These lipophilic compounds with aromatic properties can contain more than 100 different substances [3,280]. The geographic origin and the plant structures used to extract the oil determine the chemical composition of the oil, which in turn influences the antifungal and antimycotoxin activity of the plant structures [281].

Studies on the use of essential oils as biocontrol agents against aflatoxin contamination have often considered their use during food storage (notably due to their volatility). However, in vitro results can also be interpreted for potential field applications.

Table 7 lists representative results concerning the inhibition of fungal growth and aflatoxin synthesis by essential oils from various sources. Over the past decade, oregano, cinnamon, thyme, rosemary, fennel, clove, palmarosa, and eucalyptus oils have seen the most use to combat mycotoxigenic fungi and their mycotoxins [269].

In general, the results listed in Table 7 show the potential of numerous essential oils to inhibit fungal growth and/or aflatoxin production. Note that *Origanum vulgare* and *Cinnamomum zeylanicum* stimulate aflatoxin production even at low doses [294]. However, the efficacy has sometimes varied between studies involving similar essential oils, which may be attributed to differences in the chemical composition of the oils. Inhibition of fungal growth most often depends on concentration [284,299,312]. Although reduced AFB1 production is generally associated with the inhibition of fungal growth, essential oils from *Eucalyptus camaldulensis* [299] and *Cinnamomum cassia* [295], for example, effectively regulate AFB1 synthesis without inhibiting the growth of *A. flavus*. This result suggests the possibility of controlling mycotoxin contamination without disrupting the microbiota, thereby avoiding the emergence of new, potentially pathogenic species due to ecosystem alteration.

Due to their volatile compounds, essential oils may be more effective when used during food storage rather than in the field. For example, Gómez et al. (2018) [294] reported that, to inhibit fungal growth and aflatoxin production, the efficacy of essential oils from *Origanum vulgare* and *Cinnamomum zeylanicum* and their main constituents was lower in direct-contact assays than in vapor-phase tests.

The main active constituents have been identified for certain essential oils, which is favorable for their development as biocontrol agents (Table 8). Such compounds include carvacrol, carvone, cinnamaldehyde, citral, p-cymene, eugenol, limonene, menthol, linalool, vanillin, and thymol. Given their nontoxicity in mammals and their preservative potential, these compounds have been registered by the European Commission for use as flavorings in food products [315,316,317]. In some cases, essential oils have shown greater inhibitory activity when they contain higher proportions of phenolic and/or aldehyde functional groups, such as thymol, carvacrol, eugenol, and cinnamaldehyde [318,319].

### 4.3. Plant Extracts

Plant extracts are complex mixtures of diverse molecules and, depending on the extraction method and solvent used, are classified as aqueous, methanolic, chloroformic, polyphenolic, lipophilic, or ethanolic. These compounds are obtained using various techniques, including traditional extraction methods (maceration, extraction, distillation) and emerging technologies (ultrasound, microwave, electric fields, high pressure, supercritical fluids, CO_2_ extraction). The emerging technologies are designed to improve extraction quality and/or allow for the selective targeting of desired compounds [3,260]. Technologies like supercritical CO_2_ extraction are particularly appealing for field applications because they do not require using solvents, thus minimizing potential risks to plants, operators, and the environment [323].

In addition to research on essential oils, various plant extracts have been studied to determine their ability to inhibit AFB1 production by the *Aspergillus* genus and fungal growth of the same. Most studies were conducted in vitro, with the exception of one trial that was performed on maize grains [324]. Table 9 summarizes some representative results.

Most results indicate that inhibition of AFB1 synthesis is not necessarily associated with inhibition of fungal growth. Once again, the effects correlate directly with the concentrations used. One study reported notable differences in aflatoxin inhibition between *A. flavus* strains SRRC 1273 and SRRC 1299 following treatment with methanolic extracts of *Agave asperrima* and *Agave striata* flowers, suggesting that sensitivity to these extracts may vary between strains [325].

The culture medium also appears to influence the effectiveness of plant extracts. Vamvakas et al. (2021) [333] showed that the concentrations required to similarly inhibit AFB1 production are lower in a poor medium (GMS medium = 50 g/L glucose, 3 g/L (NH_4_)_2_SO_4_, 2 g/L MgSO_4_⋅7H_2_O, 10 g/L KH_2_PO_4_) than in a rich medium (YES medium = 2.0 g/L yeast extract and 150 g/L glucose).

A study on freshly harvested maize grains (water activity = 0.95) evaluated 12 methanolic or chloroform plant extracts. The results show that, although AFB1 production is less inhibited than in in vitro trials, inhibition still occurs, with no observable negative effect on germination; seedling growth was even promoted [324].

Overall, these plant extracts have positive in vitro and on-grain results, and some authors are now considering their potential for field applications [323,324,332]. Moreover, the ability of some products to inhibit AFB1 production without affecting fungal populations has been demonstrated [3]. However, identifying the bioactive compounds in these plant extracts remains necessary to develop commercial formulations [333]. Note that some bioactive compounds have already been identified, and their effects are discussed in the following section.

### 4.4. Pure Compounds

Table 10 lists the effects of certain pure compounds derived from plant extracts or essential oils.

The results demonstrate that, in in vitro conditions, numerous pure compounds can inhibit AFB1 production without affecting the growth of *Aspergillus* fungi. This result is consistent with those obtained using plant extracts.

Several studies have focused on isothiocyanates and their derivatives [277,362]. Isothiocyanates are sulfur-containing compounds produced by enzymatic conversion of glucosinolates, which occur naturally in *Brassicaceae* and related plant species.

This conversion typically occurs in response to fungal attack or tissue damage in plants. Isothiocyanates can act in dissolved or vapor form, and their specific volatility characteristics must be considered to optimize their antifungal activity. For instance, allyl isothiocyanate (AITC) is more effective in vapor form, whereas benzyl isothiocyanate is more effective in solution [277]. Various combinations have been investigated in the hope of enhancing efficacy and reducing sensory drawbacks. The AITC–cinnamaldehyde combination inhibits both AFB1 production and fungal growth, while the aromatic properties of cinnamaldehyde also mask the strong odor of AITC [342]. Manyes et al. assessed dietary exposure to AITC by potentially treating several food categories with this compound. In these experiments, all estimated daily intakes were below the acceptable daily intake of 0.02 mg/kg body weight/day established by the European Food Safety Authority [363].

A study assessing the main components of the essential oils *Origanum vulgare* and *Cinnamomum zeylanicum* revealed that these compounds retained their inhibitory effect when used individually. Note, however, that although cinnamon essential oil may stimulate AFB1 production at low concentrations, this is not the case for its main constituent, cinnamaldehyde. However, like the results with essential oils, cinnamaldehyde and carvacrol are more effective in vapor-phase assays than in direct-contact assays, limiting their suitability for field applications.

The use of pure plant-derived compounds offers the advantage of standardized effects and facilitates the elucidation of underlying mechanisms of action. Developing biocontrol agents based on well-characterized active compounds may overcome some of the major barriers to the commercialization of these compounds. Future research should focus on identifying plant extracts capable of inhibiting aflatoxin production, followed by the characterization and isolation of their bioactive constituents.

### 4.5. Limitations, Safety, and Environmental Risks Associated with Natural Substances Used as Biocontrol Agents

Certain characteristics of products derived from natural substances may limit their use as biocontrol agents in the field and must be considered.

#### 4.5.1. Formulation, Stability, and Field Persistence

Essential oils are characterized by high volatility and strong aromatic properties that can alter the organoleptic qualities of food products. As a result, they are unstable, so their application in the field would require repeated treatments, which renders widespread use difficult [3,267]. One study reported a maximum antifungal effect five days after application, which significantly decreased after 22 days [364]. Essential oils are also highly sensitive to climatic variations, which can alter their properties through oxidation, isomerization, cyclization, or dehydration reactions [3,280]. Moreover, their degradation products may be toxic [3].

Another limitation of essential oils is their poor solubility in water, which complicates field application [319]. However, this drawback may be mitigated by formulating essential oils as nano-emulsion colloidal systems containing two immiscible phases (oil and water), where one phase is dispersed as relatively small droplets (10 to 200 nm) in the other by using surface-active emulsifiers. Nano-emulsions also enhance the antifungal and antimycotoxigenic activity of essential oils [319,365,366,367].

#### 4.5.2. Phytotoxicity and Food Safety

Some essential oils may be phytotoxic at concentrations only slightly greater than those required for fungal control [3,368,369]. Phytotoxicity should therefore be evaluated for each target crop, developmental stage, formulation, and application regime under realistic greenhouse and field conditions.

In addition, essential oils can be toxic to consumers (both humans and animals), as demonstrated by several cases of poisoning following ingestion [3,370,371,372]. For food and feed applications, safety assessment should also consider residues and degradation products that remain in harvested commodities, potential dietary exposure, and possible alterations of organoleptic properties. The magnitude of these effects may depend on the administered dose, chemical composition, formulation, application method, and treatment frequency [315,316,317,363].

#### 4.5.3. Environmental Risks and Nontarget Effects

Plant extracts, particularly aqueous extracts, offer several advantages over essential oils: lower toxicity, lower impact on organoleptic properties, and simpler, more energy-efficient industrial extraction [3,373]. However, when used at higher concentrations, olfactory issues can still occur with certain isolated molecules, as observed, for example, with trans-2-hexenal [374]. Moreover, their persistence on plants requires specific formulation to prevent leaching by rain.

Natural origin should not be equated with intrinsic environmental safety. The risk assessment of essential oils, plant extracts, and purified compounds should consider their potential effects on beneficial organisms and soil microbial communities, as well as their persistence, mobility, and potential leaching into soil and water. These endpoints may depend on the dose, chemical composition, formulation, application method, and treatment frequency [315,316,317,319,368,369]. Moreover, formulations designed to improve stability and persistence, including nano-emulsions and encapsulated systems, may alter environmental exposure and should therefore be evaluated as complete formulated products rather than solely on the basis of their active substances [319,365,366,367]. Data acquired over the long term and under realistic field conditions remain limited.

#### 4.5.4. Resistance Development and Long-Term Efficacy

Information on the development of resistance to plant-derived biocontrol agents remains very limited, and this issue has rarely been evaluated under repeated-exposure or field conditions. Although the complex composition and multiple cellular targets of many essential oils and plant extracts may theoretically reduce selection pressure vis-à-vis single-site compounds [315,317], repeated exposure to sublethal concentrations, particularly of purified compounds, could favor the selection of less-sensitive fungal populations. Thus, the absence of reported resistance should not be interpreted as evidence that this risk does not exist. Instead, long-term development programs should monitor fungal sensitivity during repeated use and the integration of plant-derived agents with other preventive measures.

## 5. Commercially Available Biocontrol Agents 

Given that the use of atoxigenic strains of *A. flavus* as biocontrol agents to reduce aflatoxin contamination has been under development for several decades, numerous commercial products based on these strains are now available in various countries. The first product marketed was based on the AF36 strain and first approved by the U.S. Environmental Protection Agency (EPA) for cotton fields in Arizona and California (later extended to maize in Arizona and Texas) [48,70]. Today, AF36 Prevail is used across several states in the USA on a variety of crops, including maize, cotton, almonds, figs, and pistachios, and remains the most widely used biocontrol agent for aflatoxin mitigation worldwide [84,169,375].

Table 11 lists commercially available biocontrol agents based on atoxigenic *Aspergillus* strains, including Afla-Guard (USA), AF-X1 (Italy), and various Aflasafe products deployed across multiple African countries such as Nigeria, Ghana, Kenya, Senegal, and Tanzania. Each product contains region-specific atoxigenic strains and is tailored to local crops and environmental conditions.

Regarding plant-derived products, some essential oils have been marketed for their general antifungal properties, although not specifically for inhibiting mycotoxin production. Examples include DMC Base Natural^®^ (based on *Rosmarinus officinalis*, *Salvia officinalis*, and *Citrus* spp.) (DOMCA S.A.U, Alhendin, Spain), Cinnamite and Valero^®^ (*Cinnamomum zeylandicum*) (Mycotech Corporation, Butte, MT, USA), Sporan^®^ (*Rosmarinus officinalis*)(EcoSMART Technologies, Franklin, TN, USA), and Promax^®^ (*Thymus vulgaris*)(Bio Huma Netics, Gilbert, AZ, USA) [317]. However, the commercial development of such products for mycotoxin control remains limited due to the high variability in their chemical composition, which depends on ecological, geographical, and processing factors [317,381]. Currently, no commercial products based on plant extracts or pure plant compounds are officially marketed for mycotoxin control. Likewise, commercially available bacterial biocontrol strains are marketed for their general antifungal activity, but none makes a specific claim of mycotoxin control.

## 6. Conclusions

In conclusion, a wide range of microorganisms and plant-derived substances can reduce aflatoxin contamination by limiting the growth of aflatoxigenic fungi, competing with toxigenic strains, or directly hindering aflatoxin biosynthesis. However, the strength of the available evidence varies considerably between these approaches. Atoxigenic *A. flavus* strains currently have the strongest body of field evidence and remain the only strategy reviewed here to have reached the stage of commercial products specifically intended for aflatoxin mitigation. Although bacteria, other filamentous fungi, yeasts, essential oils, plant extracts, and purified compounds have frequently shown promise in reducing aflatoxin contamination, much of the supporting evidence still comes from in vitro studies, with comparatively fewer studies involving target matrices, controlled plant trials, or field conditions.

Each category of aflatoxin-reducing agent also offers different practical advantages. Native atoxigenic *A. flavus* strains benefit from their adaptation to local agroecosystems, their capacity to competitively exclude toxigenic strains, and their demonstrated suitability for large-scale field application. Other microbial agents, including bacteria, filamentous fungi, and yeasts, offer diverse and potentially complementary modes of action, such as competition, antibiosis, enzymatic activity, and interference with aflatoxin biosynthesis. These agents may be applied through seed treatments, soil inoculation, crop spraying, or postharvest protection. Cell-free microbial products avoid the deliberate release of live microorganisms and may facilitate dose standardization and formulation, provided that their activity and stability can be maintained. Plant-derived substances constitute a broad source of bioactive molecules: essential oils may be particularly suitable for storage or fumigation applications because of their volatility and broad antifungal activity; plant extracts may offer relatively simple production processes and, in some cases, lower toxicity and fewer effects on organoleptic properties; and purified compounds facilitate compositional standardization, controlled dosing, and mechanism-based optimization. Given that their practical suitability depends on the target crop or matrix, application stage, environmental conditions, formulation requirements, and regulatory context, these approaches should therefore be considered complementary rather than interchangeable.

Consequently, identifying a candidate with high in vitro activity should only be considered the starting point of biocontrol-agent development. Translation into practical use requires mechanistic and safety characterization, verification in relevant crops and food matrices, suitable formulation and application procedures, and multilocation and multiyear field trials that account for environmental variability. Production scalability, storage stability, persistence, regulatory requirements, and economic feasibility should also be considered early in the development process. Applying this progressive and iterative laboratory-to-field framework is essential for determining which emerging approaches can complement atoxigenic strains and contribute to reliable and sustainable management of aflatoxin contamination, particularly as climate change introduces the risk of contamination into new agricultural regions.

## Figures and Tables

**Table 1:** Examples of maximum or action levels for AFB1 and total aflatoxins in selected foods in different regions of the world.

Country	AFB1 (µg/kg)	Total Aflatoxins * (µg/kg)	Reference
European Union	- 2: cereals and cereal-derived products - 5: maize and rice intended for sorting or other physical treatment - 0.10: processed cereal-based foods and baby foods	- 4: cereals and cereal-derived products - 10: maize and rice intended for sorting or other physical treatment	[29]
United States	No separate federal action level	20: human food (*FDA action level*)	[30]
China	- 20: maize and maize products - 20: peanuts and peanut products - 0.5: cereal-based foods for infants and young children	No separate level established	[31]
Brazil	Not separately established in the cited standard	- 20: maize and maize products - 20: peanuts and peanut products - 1: cereal-based foods for infants and young children	[32]
Codex Alimentarius	Not separately established in the cited standard	- 15: maize grain intended for further processing - 10: maize flour, meal, semolina and flakes - 5: cereal-based foods for infants and young children	[33]

* Total aflatoxins correspond to the sum of AFB1, AFB2, AFG1, and AFG2. Only representative food categories are presented. The US value is an FDA action level rather than a statutory maximum level equivalent to those established in the European Union. Commodity descriptions have been abbreviated; specific exclusions, processing conditions, and sampling requirements are provided in the corresponding regulations.

**Table 7:** Studies that used essential oils to modify the development of *A. flavus* and/or the production of aflatoxin B1.

Essential Oil	Concentration	Inhibition of Fungal Development *	Inhibition of AF Production *	Reference
*Achillea millefalium* (Yarrow)	500 ppm	42% (1) 21% (3)	ND	[282]
	2000 ppm	63% (1) 48% (3)	ND	
*Achillea fragrantissima* (Yarrow)	500 ppm	16% (1) 55% (3)	ND	
	2000 ppm	84% (1) 90% (3)	ND	
*Agrimonia eupatoria* (Common agrimony)	500 ppm	45% (1) 67% (3)	ND	
	2000 ppm	82% (1) 78% (3)	ND	
*Ageratum conyzoides*	0.1 μg/mL	49% (2)	100%	[283]
	0.04 μg/mL	30%	65%	
*Allium sativum* (Garlic)	1%	2.2 to 15.9% (2)	ND	[284]
	4%	26.6 to 49.8% (2)	28.5 to 62.8%	
*Allium sativum* (Garlic)	2% in stored wheat grains	100% (1)	85.4%	[285]
*Amomum subulatum* (Cardamom)	0.25 mg/mL	25% (1)	69%	[286]
	0.5 mg/mL	74% (1)	100%	
*Azadirachta indica* (Neem)	6–7 µg/mL	100% (1)	77%	[287]
*Boswellia sacra*	10 μg/mL	16.60% (1)	50%	[288]
	40 μg/mL	83.30% (1)	83.30%	
*Calendula officinalis*	500 ppm	35% (1) 6% (3)	ND	[282]
	2000 ppm	90% (1) 93% (3)	ND	
*Callistemon lanceolatus*	0.546 mg/mL	79.60% (1)	100%	[289]
	0.819 mg/mL	100% (1)	100%	
*Carum carvi* (Chitosan nanoemulsion)	0.4–0.5 µL/mL	100% (1)	100%	[290]
*Cedrus atlantica* (Cedar)	500 ppm	31% (1)	ND	[291]
	2000 ppm	61% (1)	ND	
Cinnamon	1.00 µL/mL	54.3% (1)	81.7%	[292]
*Cinnamomum jendenianum* (Cinnamon)	4 μL/mL	63% (1)	84%	[293]
	6 μL/mL	84% (1)	100%	
*Cinnamomum jendenianum* (Cinnamon)	1%	23.4 to 41.6% (2)	ND	[284]
	4%	50.4 to 72.7% (2)	59.2 to 78.5%	
*Cinnamomum zeylanicum* (Cinnamon)	295 to 560 mg/L	50% (1)	ND	[294]
	>1000 mg/L	90% (1)	ND	
	1000 mg/L	ND	100%	
*Cinnamomum verum* (Cinnamon)	500 ppm	85% (1)	ND	[291]
	2000 ppm	100% (1)	ND	
*Cinnamomum verum* (Cinnamon)	0.125% in wheat	75% (1)	73.8%	[285]
*Cinnamomum verum* (Cinnamon bark)	1 µg/mL	ND (1)	>70%	[287]
*Cinnamomum zeylanicum* (Cinnamon)	500 ppm	94% (1) 81% (3)	ND	[282]
	2000 ppm	100% (1) 100% (3)	ND	
*Cinnamomum cassia* (Cinnamom)	4% (*v*/*v*)	0% (1)	96%	[295]
*Citrus sinendis* (Orange)	500 ppm	27% (1)	ND	[291]
	2000 ppm	60% (1)	ND	
*Coriandrum sativum* (Coriander)	0.75 μL/mL	66.50% (1)	25%	[296]
*Cuminum cyminum* (Cumin)	0.4 μL/mL	52% (1)	67%	[267]
	0.5 μL/mL	91% (1)	100%	
	0.6 µL/mL	100% (1)	100%	
*Cuminum cyminum* (Cumin)	500 ppm	44% (1)	ND	[291]
	2000 ppm	66% (1)	ND	
*Curcuma longa*	0.5% (*v*/*v*)	ND (1)	99%	[297]
*Cymbopogon citratus* (Lemongrass)	0.1 mg/mL	14% (1)	75%	[298]
	0.2 mg/mL	3% (1)	100%	
	0.5 mg/mL	89% (1)	100%	
*Cymbopogon citratus* (Lemongrass)	500 ppm	85% (1)	ND	[291]
	2000 ppm	100% (1)	ND	
*Cymbopogon nardus* (Lemongrass)	0.25 µL/mL	5% (1)	62%	[299]
	1 µL/mL	67% (1)	100%	
*Cymbopogon winterianus* (Lemongrass)	500 ppm	54% (1)	ND	[291]
	2000 ppm	94% (1)	ND	
*Cymbopogon schoenanthus* (Camel grass)	0.25 µL/mL	19% (1)	81% (1)	[299]
	0.5 µL/mL	46% (1)	99% (1)	
*Cymbopogon martini* (Palmarosa)	0.3 μL/mL	50% (1)	80%	[300]
	0.4 μL/mL	95% (1)	100%	
*Elettaria cardamomum* (Cardamom)	500 ppm	48% (1)	ND	[291]
	2000 ppm	81% (1)	ND	
*Eucalyptus* vapor	MIC 0.05–0.31 µL/mL	100% (1)	100%	[301]
*Eucalyptus camaldulendis*	0.25 µL/mL	2% (1)	47% (1)	[299]
	1 µL/mL	23% (1)	97% (1)	
*Eucalyptus globulus*	500 ppm	49% (1)	ND	[291]
	2000 ppm	83% (1)	ND	
*Foenicculum vulgare* (Fennel)	0.75 μL/mL	54.40% (1)	23%	[296]
*Laurus nobilis* (Laurel)	5% (*v*/*v*)	36.40% (1)	46%	[295]
*Lavandula dentata* (Lavender)	500 ppm	49% (1)	ND	[291]
	2000 ppm	64% (1)	ND	
*Lippia rugosa* (Verbena)	600 μg/mL	5% (1)	61%	[302]
	1000 μg/mL	5% (1)	100%	
*Matricaria chamomillal* *(Chamomile)*	500 ppm	35% (1) 68% (3)	ND	[282]
	2000 ppm	86% (1) 86% (3)	ND	
*Mentha spicata* (Mint)	0.6 μL/mL	66.40% (1)	50.20%	[267]
	0.9 μL/mL	84.90% (1)	100%	
*Mentha spicata* (Mint)	1%	15.4 to 44.0% (2)	ND	[284]
	4%	29.6 to 59.2% (2)	46.1 to 71.9%	
*Mentha spicata* (Mint)	500 ppm	49% (1)	ND	[291]
	2000 ppm	85% (1)	ND	
*Mentha viridis* (Mint)	500 ppm	52% (1) 76% (3)	ND	[282]
	2000 ppm	95% (1) 92% (3)	ND	
*Mentha piperita* (Peppermint)	500 ppm	30% (1)	ND	[291]
	2000 ppm	55% (1)	ND	
*Mentha piperita* (Peppermint)	2% in stored wheat grains	100% (1)	81.7%	[285]
*Mentha piperita* (Peppermint)	300 mg/kg	ND (2)	85 to 90%	[303]
*Minthostachys verticillate* (Peperina)	300 mg/kg	ND (2)	85 to 90%	
*Ocimum sanctum* (Basil)	0.1 μg/mL	72.50% (1)	88.40%	[304]
	0.2 μg/mL	90.10% (1)	100%	
*Ocimum basilicum* (Sweet basil)	4% (*v*/*v*)	60% (1)	52%	[295]
*Ocimum basilicum* (Sweet basil)	500 ppm	30% (1) 71% (3)	ND	[282]
	2000 ppm	87% (1) 88% (3)	ND	
*Ocimum tenuiflorum* (Holy basil)	0.75–1.5 µL/mL	100% (1)	100%	[305]
*Ocimum basilicum* (Sweet basil)	1.25–2.0 µL/mL	100% (1)	100%	
*Ocimum gratissimum* (Clove basil)	1.25–2.5 µL/mL	100% (1)	100%	
*Origanum majoricum* (Oregano)	100 μL/mL	40% (1)	25.6%	[63]
	500 μL/mL	100% (1)	100%	
*Origanum vulgare* (Oregano)	100 μL/mL	3% (1)	26.8%	
	500 μL/mL	60% (1)	94.2%	
*Origanum virens* (Oregano)	350 μL/mL	ND (1)	7.3%	[306]
	1000 μL/mL	ND (1)	78.1%	
	10 μL/mL	5% (1)	2.30%	[63]
	100 μL/mL	100% (1)	99.70%	
*Origanum vulgare* (Oregano)	300 mg/kg	ND (2)	100%	[303]
*Origanum vulgare* (Oregano)	152 to 505 mg/L	50% (1)	ND	[294]
	820 to >1000 mg/L	90% (1)	ND	
	1000 mg/L	ND (1)	100%	
*Origanum vulgare* (Oregano emulsion)	0.15–0.6 mg/mL	100% (1)	100%	[307]
*Oregano–thyme* (Emulsion)	0.15–0.6 mg/mL	100% (1)	100%	
*Peumus boldus* (Boldo)	500 mg/kg	80% (2)	ND	[303]
*Pimpinella anisum* (Anise)	500 mg/kg	80% (2)	100%	
*Piper betle* (Betel)	0.4 μL/mL	26.70% (1)	74.50%	[308]
	0.6 μL/mL	77.80% (1)	100%	
*Piper nigrum* (Black pepper)	500 ppm	42% (1)	ND	[291]
	2000 ppm	64% (1)	ND	
*Romarinus officinalis* (Rosemary)	10 μL/mL	0% (1)	52%	[63]
	500 μL/mL	0% (1)	90%	
*Romarinus officinalis* (Rosemary)	5 µL/disk	32.80% (3)	ND	[268]
	20 µL/disk	64.30% (3)	ND	
	250 ppm	6% (3)	2%	
	450 ppm	0% (3)	100%	
*Romarinus officinalis* (Rosemary)	1%	14.1 to 36.8% (2)	ND	[284]
	4%	44.4 to 61.1% (2)	38.6 to 64.4%	
*Satureja montana* (Savory)	350 μL/mL	ND (1)	84.70%	[306]
	1000 μL/mL	ND (1)	86.30%	
	10 μL/mL	20% (1)	23%	[63]
	100 μL/mL	100% (1)	100%	
*Satureja khozistanica* (Savory)	62.5 mg/L	12% (1)	48%	[309]
	250 mg/L	63% (1)	88%	
	375 mg/L	100% (1)	100%	
*Satureja macrosiphonia* (Savory)	250 mg/L	28% (1)	32%	
	375 mg/L	46% (1)	44%	
	500 mg/L	65% (1)	67%	
*Syzygium aromaticum* (Clove)	500 ppm	100% (1)	10%	[291]
	2000 ppm	100% (1)	46%	
*Syzygium aromaticum* (Clove)	0.25–0.5 µg/mL	100% (1)	>50–100%	[287]
*Syzygium aromaticum* (Clove)	2% stored wheat grains	100% (1)	94.8%	[285]
*Syzygium aromaticum* (Clove)	1.00 µL/mL	ND (1)	73.4%	[292]
*Syzygium aromaticum*	0.2–0.4 µL/mL	100% (1)	100%	[310]
*Syzygium aromaticum* (Nanoemulsified)	0.15–0.35 µL/mL	100% (1)	100%	
*Thymus vulgaris* (Thyme)	1.00 µL/mL	ND	65.2%	[292]
*Thymus vulgaris* (Thyme emulsion)	0.15–0.6 mg/mL	ND (1)	ND	[307]
*Thymus vulgaris* (Thyme)	0.3 μL/mL	46.90% (1)	75.60%	[311]
	0.7 μL/mL	100% (1)	100%	
	10 μL/mL	5% (1)	35%	[63]
	500 μL/mL	8% (1)	100%	
*Thymus daenendis* (Thyme)	62.5 mg/L	3% (1)	11%	[309]
	250 mg/L	57% (1)	87%	
	375 mg/L	100% (1)	100%	
*Thymus daenendis* (Thyme)	1%	22.4 to 53.6% (2)	ND	[284]
	4%	44.2 to 70.6% (2)	53.5 to 86.0%	
*Thymus vulgaris* (Thyme)	50 µg/mL	0% (1)	64%	[312]
	150 µg/mL	0% (1)	100%	
	250 µg/mL	MIC (1)	100%	
*Thymus vulgaris* (Thyme)	500 ppm	89% (1)	ND	[291]
	2000 ppm	100% (1)	ND	
*Thymus vulgaris* (Thyme)	125 ppm	88% (1) 74% (3)	ND	[282]
	250 ppm	100% (1) 85% (3)	ND	
*Thymus vulgaris* (Thyme vapor)	0.025–0.1 µL/mL	100% (1)	100%	[301]
*Trachyspermum ammi* (Ajwain)	0.4–0.5 µL/mL	100% (1)	100%	[313]
*Trachyspermum copticum*	5 µL/disk	52.80% (3)	ND	[268]
	20 µL/disk	100% (3)	ND	
	350 ppm	0% (3)	83%	
	450 ppm	0% (3)	90%	
*Zingiber officinale* (Ginger)	6–7 µg/mL	100% (1)	60%	[287]
*Zingiber officinale* (Ginger)	500 ppm	8% (1)	ND	[291]
	2000 ppm	36% (1)	ND	
*Zingiber officinale–Trachyspermum ammi–Coleus aromaticus* (formulation)	0.3–0.4 µL/mL	100% (1)	100%	[314]

ND = not described, MIC = minimal inhibitory concentration preventing macroscopic development of *A. flavus*. (1) Effect on *A.flavus*; (2) effect on *A. flavus* and *A. parasiticus*; (3) effect on *A. parasiticus*. * Inhibition of fungal development or AF production is expressed in % compared to a control culture not exposed to the essential oil being tested.

**Table 8:** Composition of some essential oils that hinder aflatoxin production.

Essential Oil	Main Bioactive Compounds	References
*Cuminum cyminum* (Cumin)	Cymene (47.08%)	[267]
*Cymbopogon citratus* (Lemongrass)	Geranial (37.71%), neral (33.17%), myrcene (11.41%)	[320]
*Mentha spicata* (Spearmint)	Carvone (59.6%)	[321]
*Rosmarinus officinalis* (Rosemary)	Piperitone (23.65%), α-pinene (14.94%), limonene (14.89%), 1,8-cineol (7.43%)	[268]
*Trachyspermum copticum* (Ajowan)	Thymol (37.2%), p-cymene (32.3%), γ-terpinene (27.3%)	
*Syzygium aromaticum* (Clove)	Eugenol (83.25%), E-caryophyllene (13.36%), α-humulene (2.18%)	[291]
	Eugenol (12.15 mg/g)	[322]
*Cinnamomum zeglanicum* (Cinnamon)	Cinnamaldehyde (25.74 mg/g)	
*Thymus daenensis* (Thyme)	Thymol (14.49 mg/g), carvacrol (1.17 mg/g)	
*Ocimum basilicum (Sweet basil)*	Linalool (25.40 %), tridecanedial (14.12%), geranial (10.21%)	[305]
*Origanum vulgare* (Oregano)	Carvacrol	[294]
*Cinnamomum zeylanicum* (Cinnamon)	Cinnamaldehyde	

**Table 9:** In vitro effect of plant extracts on the inhibition of *A. flavus* growth and aflatoxin B1 synthesis.

Plant	Extraction Solvent	Concentration	Inhibition of *A. flavus* Growth *	Inhibition of AFB1 Production ^#^	Reference
*Agave asperrima* (Agave)	Methanol	0.125 mg/mL	11%	35%	[325]
		0.25 mg/mL	3 to 42%	16 to 98%	
*Agave striata* (Agave)	Methanol	0.375 mg/mL	0%	60%	
		0.75 mg/mL	30%	99%	
*Acacia catechu* (Acacia)	Methanol	2 mg/mL	26.20%	80% (57% on grains)	[324]
*Acacia ferruginea* (rusty acacia)			23.80%	74.6% (54% on grains)	
*Adenanthera pavonine* (Lucky red seeds)	Chloroform		19.6%	61.6% (34% on grains)	
*Albizia amara*			59%	100% (87% on grains)	
*Albizia odoratissima* (Black siris)	Methanol		22.6%	70% (51% on grains)	
*Albizia saman* (Bitter albizia)			57.8%	100% (87% on grains)	
*Anogeissus latifolia* (Axlewood)			27.8%	83.3% (64% on grains)	
*Caesalpinia coriaria* (Divi-divi)			18.6%	70% (54% on grains)	
*Cassia spectabilis* (Golden wonder tree)	Chloroform		42.4%	93.3% (79% on grains)	
*Dodonaea viscosa* (Hopbush)	Methanol		14.3%	66% (29% on grains)	
*Prosopis juliflora* (Mesquite)			22.2%	73.3% (49% on grains)	
*Solanum indicum* (Indian nightshade)	Chloroform		63.5%	100% (99% on grains)	
*Allium sativum* (Garlic)	50% ethanol-water	12.5 mg/mL	96.35%	72.76%	[326]
		25 mg/mL	97.08%	76.26%	
		50 mg/mL	98.05%	74.98%	
	Water	12.5 mg/mL	53.82%	66.12%	
		25 mg/mL	61.78%	62.68%	
		50 mg/mL	88.76%	62.08%	
	Acetone	12.5 mg/mL	63.73%	67.44%	
		25 mg/mL	65.92%	69.79%	
		50 mg/mL	89.24%	65.55%	
	Ethyl acetate	12.5 mg/mL	63.52%	70.68%	
		25 mg/mL	61.82%	68.89%	
		50 mg/mL	88.92%	69.85%	
	Ethanol	12.5 mg/mL	79.78%	67.80%	
		25 mg/mL	81.92%	69.36%	
		50 mg/mL	89.24%	68.20%	
*Aloysia citrodora* (Lemon verbena)	Water	0.04 mg DM/mL	NR	59%	[327]
		0.08 mg DM/mL	NR	63%	
		0.15 mg DM/mL	NR	77%	
		0.30 mg DM/mL	2.14%	87%	
*Aloysia citrodora* (Lemon verbena)	Water	6 mg DM/mL	2%	78%	[62]
*Annona muricata* (Soursop)		6 mg DM/mL	2%	69%	
*Dysphania ambrosioides* (Mexican tea)		6 mg DM/mL	2%	47%	
*Minthostachys mollis* (Andean mint)		6 mg DM/mL	3%	89%	
*Myrciaria dubia* (Camu-camu)		6 mg DM/mL	1%	32%	
*Uncaria tomentosa* (Cat's claw)		6 mg DM/mL	1%	58%	
*Annona muricata* (Soursop)	Water	0.15 mg DM/mL	0%	20%	[328]
		0.08 mg DM/mL	1%	25%	
		0.04 mg DM/mL	2%	35%	
		0.30 mg DM/mL	3%	53%	
*Uncaria tomentosa* (Cat's claw)	Water	0.15 mg DM/mL	1%	10%	
		0.08 mg DM/mL	1%	22%	
		0.04 mg DM/mL	2%	33%	
		0.30 mg DM/mL	2.5%	51%	
*Azadirachta indica* (Neem)	Methanol	75 mg/mL	81.8%	81.25%	[329]
	Ethanol	75 mg/mL	84.1%	89.3%	
	Chloroform	75 mg/mL	63.6%	63.4%	
	Ethyl acetate	75 mg/mL	56.8%	57.1%	
*Azadirachta indica* (Neem)	Ethanol	31.25 mg/mL	45%	NR	[330]
	Methanol	250 mg/mL	NR	>90%	
	Ethanol	250 mg/mL	NR	>90%	
*Azadirachta indica* (Neem)	70% ethanol	10 μg/mL	NR	54.6%	[287]
*Cinnamomum verum* (Ceylon cinnamon)		7.0 μg/mL	NR	65.6%	
		7.5 μg/mL	100%	100%	
*Syzygium aromaticum* (Clove)		4.0 μg/mL	NR	35.6%	
		4.5 μg/mL	100%	100%	
*Zingiber officinale* (Ginger)		10 μg/mL	NR	26.6%	
*Camellia sinensis* (Tea plant)	70% methanol	2 mg/mL	100%	89%	[331]
*Cistus incanus* (Rockrose)	Methanol	0.2 g/mL	NR	87.1 to 90.1% (1)	[332]
*Micromeria graeca* (Hysope)	Water	0.078 mg/mL	0%	28.5%	[277]
		0.625 mg/mL	0%	52%	
		10 mg/mL	0%	99%	
*Vaccinium myrtillus* (Blueberry)	Water	0.17 ppm	0%	50% (nutrient-poor medium)	[333]
		0.27 ppm	0%	80% (nutrient-poor medium)	
		1.3 ppm	0%	50% (nutrient-rich medium)	
		1.8 ppm	0%	80% (nutrient-rich medium)	
	Methanol	2.8 ppm	0%	50% (nutrient-poor medium)	
		3.3 ppm	0%	50% (nutrient-rich medium)	
		3.7 ppm	0%	80% (nutrient-poor medium)	
		4.8 ppm	0%	80% (nutrient-rich medium)	
*Sonchus oleraceus* (Common sowthistle)	Water	1.5 mg/mL	NR	26%	[334]
		5 mg/mL	NR	78%	
*Cichorium pumilum* (Wild endive)		1.5 mg/mL	NR	19%	
		5 mg/mL	NR	69%	
*Portulaca oleracea* (Purslane)		1.5 mg/mL	NR	32%	
		5 mg/mL	NR	82%	
*Fagopyrum esculentum* (Buckwheat)	Polyphenol (rich fraction)	50 ng/mL	NR	92%	[323]
		100 ng/mL	37%	93%	
	Lipophilic fraction	10 μg/mL	74%	67%	
		100 μg/mL	NR	89%	
*Melia azedarach* (Chinaberry)	Water	125 mg/mL	51%	68%	[335]
		500 mg/mL	73%	76%	
*Mimosa tenuiflora* (Black jurema)	Water	0.025 mg DM/mL	0%	10%	[279]
		0.05 mg DM/mL	0%	20%	
		0.1 mg DM/mL	0%	30%	
		0.15 mg DM/mL	2%	50%	
		0.20 mg DM/mL	3%	66%	
*Ocimum basilicum* (Sweet basil)	Ethanol	15.75 mg/mL	40%	44.4%	[336]
		250 mg/mL	85%	83.3%	
	Chloroform	250 mg/mL	75%	78.9%	
	n-Hexane	250 mg/mL	70%	73.3%	
*Origanum onites* (Turkish oregano)	Ethanol	20 mg/mL	79.3%	100%	[337]
		25 mg/mL	100%	100%	
*Thymus capitatus* (Conehead thyme)		20 mg/mL	59.9%	98.96%	
		25 mg/mL	100%	100%	
*Origanum vulgare* (Oregano)	Water	0.25 mg/mL	100%	NR	[338]
		1 mg/mL	NR	90.46%	
		2 mg/mL	NR	95.20%	
		4 mg/mL	NR	98.68%	
		8 mg/mL	NR	100%	
*Urginea epigea* (Squill)	Methanol	50 mg/mL	100%	NR	[339]
*Vitis vinifera* (Grapevine)	30% ethanol-water	0.5 g/L	NR	97%	[340]
		1.0 g/L	NR	98%	
		2.5 g/L	NR	94%	
		5.0 g/L	NR	100%	
		15.0 g/L	92%	100%	
*Thymus daenensis* (Thyme)	Ethanol	1 mg/mL	20%	52%	[309]
		4 mg/mL	100%	97%	
	Ethanol-water	1 mg/mL	20%	52%	
		4 mg/mL	90%	97%	
	Water	1 mg/mL	20%	11%	
		6 mg/mL	65%	61%	
*Satureja khozistanica* (Khuzestan savory)	Ethanol	1 mg/mL	73%	72%	
		4 mg/mL	100%	100%	
	Ethanol-water	1 mg/mL	51%	50%	
		4 mg/mL	65%	100%	
	Water	1 mg/mL	14%	6%	
		6 mg/mL	63%	48%	
*Satureja macrosiphonia* (Savory)	Ethanol	0.5 mg/mL	5%	20%	
		6 mg/mL	88%	75%	
	Ethanol-water	0.5 mg/mL	5%	20%	
		6 mg/mL	78%	78%	
	Water	0.5 mg/mL	5%	20%	
		6 mg/mL	60%	78%	

(1) *A. parasiticus*; NR: not reported. * Fungal growth refers to the growth-related endpoint evaluated in the original study, such as radial or mycelial growth or fungal biomass. Percentages represent inhibition relative to an untreated control. **^#^**: expressed in % compared with an untreated culture.

**Table 10:** A selection of plant-derived and essential-oil-derived compounds used to inhibit *A. flavus* growth and/or aflatoxin B1 production in vitro.

Molecule	Concentration	Inhibition of *A. flavus* Growth *	Inhibition of AFB1 Production ^#^	Reference
Allyl-isothiocyanate	34.7 µg/mL	NR	50%	[341]
	86.2 μg/mL	50%	NR	
	0.076 mM	50% (1)	NR	[342]
	0.125 µL/L	0%	NR	[278]
	1 µL/L	100%	NR	
	5 µL/L	100%	NR	
	60 μg/mL	NR	50%	[343]
	100 μg/mL	NR	70%	
	175 μg/mL	50%	about 80%	
Ascorbic acid	12 mM	0%	100%	[344]
Benzyl-isothiocyanate	5.4 µg/mL	NR	50%	[341]
	15.2 μg/mL	50%	NR	
Caffeic acid	12 mM	0%	95%	[345]
Caffeic acid	1000 µg/mL	0%	>92% (>74% (1)) (2)	[346]
	2000 µg/mL	0%	>92% (>76% (1)) (2)	
Carvacrol	98 to 145 mg/L	50%	NR	[294]
	770 to 860 mg/L	90%	NR	
	250 mg/L	NR	100%	
Chlorogenic acid	12 mM	0%	92%	[344]
Cinnamaldehyde	52 mg/L	24.4%	38%	[347]
	104 mg/L	100%	100%	
Cinnamaldehyde	0.4 mM	0%	68.9%	[348]
	0.8 mM	100%	100%	
Cinnamaldehyde	0.246 mM	50% (1)	NR	[342]
Cinnamaldehyde	49 to 52.6 mg/L	50%	NR	[294]
	89.7 to 90.5 mg/L	90%	NR	
	100 mg/L	NR	100%	
Cinnamaldehyde + linalool Pickering emulsion	32 μL/mL (MIC)	100%	NR	[349]
	4% oil phase (CN:LN 4:2) (2)	65.75%	78.63%	
Citral	0.56 mM	0%	41.8%	[348]
	2.8 mM	100%	100%	
Curcumin	0.1 mL/L	49.2%	NR	[297]
	0.5 mL/L	96%	NR	
6,7-Dimethoxycoumarin	10.82 µg/mL	18% (1)	33%	[350]
	16.24 µg/mL	43% (1)	56%	
Eugenol	0.48 mM	0%	65%	[348]
	0.8 mM	0%	95.4%	
Eugenol	0.05 mM	0%	30.9%	[351]
	0.5 mM	11.4%	100%	
Eugenol in chitosan coating	ND (2)	84.16%	95.36%	[352]
Ferulic acid	10 mM	0%	NR	[353]
	30 mM	100%	100%	
Gallic acid	0.8 g/L	20%	100%	[354]
Gallic acid	12 mM	0%	80%	[344]
Hesperidin	0.15 mM	NR	80%	[355]
	0.30 mM	NR	91%	
Methyl-isothiocyanate	50 μg/mL	NR	50%	[343]
	62 μg/mL	50%	about 67%	
	70 μg/mL	NR	100%	
	17.4 µg/mL	NR	50%	[341]
	43.8 μg/mL	50%	NR	
Methyl Syringate	0.5 mM	0%	40%	[356]
Neocryptolepine	80 μg/mL	63%	80%	[357]
Naringin	0.15 mM	NR	65%	[262]
	0.30 mM	NR	70%	
Neo-hesperidin	0.15 mM	NR	86%	
	0.30 mM	NR	68%	
Piperine	0.0006 mM	0%	30%	[358]
	0.17 mM	35%	100%	
Piperine	0.7 mg/mL	40%	75%	[359]
Piperlongumine	0.2 mg/mL	15%	96%	
Piperoctadecalidine	0.7 mg/mL	47%	100%	
Piperonaline	0.7 mg/mL	30%	70%	
Phenyl-ethyl-isothiocyanate	11.6 µg/mL	NR	50%	[341]
	21.4 μg/mL	50%	NR	
Scopoletin	2 mM	25%	100%	[360]
Trans-cinnamic acid	10 mM	0%	NR	[353]
	25 mM	100%	100%	
3,6,7-Trihydroxy-tetralone	25 µg/mL	NR	48%	[361]
	100 µg/mL	38.34%	99%	
Vanillic acid	1000 µg/mL	0%	>88% (>89% (1)) (2)	[346]
	2000 µg/mL	0%	>91% (>92% (1)) (2)	

(1) Assay on *A. parasiticus*; (2) assay on grains or a grain-based food matrix; NR = not reported. * Fungal growth refers to the growth-related endpoint evaluated in the original study, such as radial or mycelial growth or fungal biomass. Percentages represent inhibition relative to an untreated control. **^#^**: expressed in % compared with an untreated culture.

**Table 11:** Commercial biocontrol agents based on nonaflatoxigenic *Aspergillus* strains authorized on the market.

Name	Strains	Country	Authorized Crops	References
*A. flavus* AF36 Prevail*^®^* (Arizona Cotton Research & protection council, Phenix, AZ, USA)	AF36	USA	Cotton, maize, pistachio, almonds, figs	[376]
*A. flavus AF36 Prime^®^* (Arizona Cotton Research & protection council, Phenix, AZ, USA)	AF36	USA	Cotton, maize, pistachio, almonds, figs	[377]
Afla-Guard^®^ (Syngenta crop protection, Greensboro, NC, USA)	NRRL21882	USA	Maize and peanuts	[376,378]
AF-X1^®^ (Pioneer Hi-Breed Italia, Gadesco Pieve, Delmona, Italy)	MUCL54911	Italy	Maize	[70]
Aflasafe SN01 (BAMTAARE SA, Kahone, Senegal)	M2-7, M21-11, Ms14-19, Ss19-14	Senegal and Gambia	Maize and peanuts	[150]
Aflasafe GH01 (CalliGhana, Accra, Ghana)	GHG079-4, GHG083-4, GHG321-2, GHM174-1	Ghana	Maize, peanuts, sorghum	[140]
Aflasafe GH02 (CalliGhana, Accra, Ghana)	GHM511-3, GHM109-4, GHM001-5, GHM287-10			
Aflasafe™ (Harvestfiled Industries Limited, Lagos, Nigeria)	Ka16127, La3279, La3304, Og0222	Nigeria	Maize and peanuts	[256]
Aflasafe KE01 (Kenya Agricultural and Livestock Research Organization, Katumani, Machakos, Kenya)	C6-E, C8-F, E63-I, R7-H	Kenya	Maize	
Aflasafe BF01 (BAMTAARE SA, Kahone, Senegal)	M011-8, G018-2, M109-2, M110-7	Burkina Faso	Maize and peanuts	[379]
Aflasafe TZ01 (A to Z Textile Mills Ltd, Arusha, Tanzania)	TMS199-3, TMH104-9, TGS364-2, TMH 30-8	Tanzania	Maize and peanuts	[380]
Aflasafe TZ02 (A to Z Textile Mills Ltd, Arusha, Tanzania)	TMS64-1, TGS55-6, TMS205-5, TMS137-3			
Aflasafe ZM01 (Aflalivre Moçambique SA, Nampula, Mozambique)	110MS-05, 38MS-03, 46MS-02, 03MS-10	Zambia		
Aflasafe ZM02 (Aflalivre Moçambique SA, Nampula, Mozambique)	31MS-12, 12MS-10, 47MS-12, 64MS-03			
Aflasafe MW02 (The Anchorage Group, Lilonwe, Malawi)	MW258-6, MW332-10, MW248-11, MW204-7	Malawi		
Aflasafe MWMZ01 (The Anchorage Group, Lilonwe, Malawi)	MW199-1, MW097-8, MW246-2, MW238-2			
Aflasafe MZ02 (Aflalivre Moçambique SA, Nampula, Mozambique)	GP5G-8, MZG071-6, MZM028-5, MZM250-8	Mozambique		
Aflasafe MWMZ01 (Aflalivre Moçambique SA, Nampula, Mozambique)	GP5G-8, GP1H-12, MZM594-1, MZM029-7			
Aflasafe MZ02 (Aflalivre Moçambique SA, Nampula, Mozambique)	GP5G-8, MZG071-6, MZM028-5, MZM250-8	Mozambique	Maize and peanuts	[165]

## Data Availability

No new data were created or analyzed in this study.

## Abbreviations

The following abbreviations are used in this manuscript:

AF	Aflatoxin(s)
AFB1	Aflatoxin B1
AFB2	Aflatoxin B2
AFG1	Aflatoxin G1
AFG2	Aflatoxin G2
AFM1	Aflatoxin M1
AFM2	Aflatoxin M2
AFPs-WYH	Antifungal peptide fraction produced by *Lactiplantibacillus plantarum* WYH
AITC	Allyl isothiocyanate
ATP	Adenosine triphosphate
CFS	Cell-free supernatant
CPA	Cyclopiazonic acid
DALYs	Disability-adjusted life years
DM	Dry matter
EPA	United States Environmental Protection Agency
EPS	Exopolysaccharide(s)
FAO	Food and Agriculture Organization of the United Nations
FDA	United States Food and Drug Administration
GAP	Good agricultural practices
GFP	Green fluorescent protein
GMS	Glucose-mineral salts medium
LAB	Lactic acid bacteria
MIC	Minimum inhibitory concentration
ND	Not described
NR	Not reported
PDA	Potato dextrose agar
ROS	Reactive oxygen species
TLC	Thin-layer chromatography
US	United States
USA	United States of America
UV	Ultraviolet
VCG	Vegetative compatibility group
VOCs	Volatile organic compounds
YES	Yeast extract-sucrose medium
YMA	Yeast extract-malt extract agar
